# Photobiont Diversity in Lichen Symbioses From Extreme Environments

**DOI:** 10.3389/fmicb.2022.809804

**Published:** 2022-03-29

**Authors:** Roberto De Carolis, Agnese Cometto, Patricia Moya, Eva Barreno, Martin Grube, Mauro Tretiach, Steven D. Leavitt, Lucia Muggia

**Affiliations:** ^1^Department of Life Sciences, University of Trieste, Trieste, Italy; ^2^Botánica, ICBIBE, Faculty of CC. Biológicas, Universitat de València, Valencia, Spain; ^3^Institute of Biology, University of Graz, Graz, Austria; ^4^Department of Biology, Brigham Young University, Provo, UT, United States

**Keywords:** chloroplast morphology, culture, phylogeny, *Rhizoplaca*, *Tephromela*, *Trebouxia*

## Abstract

Fungal–algal relationships—both across evolutionary and ecological scales—are finely modulated by the presence of the symbionts in the environments and by the degree of selectivity and specificity that either symbiont develop reciprocally. In lichens, the green algal genus *Trebouxia* Puymaly is one of the most frequently recovered chlorobionts. *Trebouxia* species-level lineages have been recognized on the basis of their morphological and phylogenetic diversity, while their ecological preferences and distribution are still only partially unknown. We selected two cosmopolitan species complexes of lichen-forming fungi as reference models, i.e., *Rhizoplaca melanophthalma* and *Tephromela atra*, to investigate the diversity of their associated *Trebouxia* spp. in montane habitats across their distributional range worldwide. The greatest diversity of *Trebouxia* species-level lineages was recovered in the altitudinal range 1,000–2,500 m a.s.l. A total of 10 distinct *Trebouxia* species-level lineages were found to associate with either mycobiont, for which new photobionts are reported. One previously unrecognized *Trebouxia* species-level lineage was identified and is here provisionally named *Trebouxia* “A52.” Analyses of cell morphology and ultrastructure were performed on axenically isolated strains to fully characterize the new *Trebouxia* “A52” and three other previously recognized lineages, i.e., *Trebouxia* “A02,” *T. vagua* “A04,” and *T. vagua* “A10,” which were successfully isolated in culture during this study. The species-level diversity of *Trebouxia* associating with the two lichen-forming fungi in extreme habitats helps elucidate the evolutionary pathways that this lichen photobiont genus traversed to occupy varied climatic and vegetative regimes.

## Introduction

Lichens are self-sustaining ecosystems formed by the interaction of an exhabitant fungus and an extracellular arrangement of one or more microscopic photosynthetic partners and an indeterminate number of other microorganisms (Hawksworth and Grube, 2020). The symbiotic phenotype of a lichen, however, is thought to be mainly dictated by the phenotype of the predominant exhabitant lichen-forming fungus, i.e., the mycobiont (Honegger, 2012; Hawksworth and Grube, 2020). The photosynthetic partners, instead, consist of either unicellular green microalgae or blue-green cyanobacteria, i.e., chlorobionts or cyanobionts, respectively (the biologically relevant photobiont, Paul et al., 2018). The obligate symbiotic relationship established between the mycobiont and the photobionts represents one of the most successful nutritional strategies among fungi, occurring in almost every terrestrial environment on Earth (Lücking et al., 2016).

The success of these fungal–algal relationships both across evolutionary and ecological scales is finely modulated by the two main symbionts and their degree of selectivity and specificity that they develop reciprocally (Beck et al., 2002; Yahr et al., 2004, 2006). Thus, specific or generalist associations among the lichen symbionts (Beck et al., 2002; Miadlikowska et al., 2006; Leavitt et al., 2015; Grube et al., 2016; Chagnon et al., 2019) have significant impacts on the structure of lichen communities and species distribution (Muggia et al., 2014b; Werth and Sork, 2014; Steinova et al., 2019). Some mycobionts tend to accept only single algal lineages, while others, more generalist, can associate with many different algal lineages (Yahr et al., 2004). Similarly, photobionts and their preference toward the fungal partners have also been reported (Peksa and Škaloud, 2011).

The genus *Trebouxia* Puymaly is one of the most frequently occurring chlorobiont in lichens. Its species associate with mycobionts which are phylogenetically distantly related among Ascomycota and come from very diverse ecological conditions (Nelsen et al., 2021). While *Trebouxia* has received considerable attention, only recently has species diversity been more fully recognized and characterized according to their genetic, and to a less extent morphological, diversity and ecological preferences (Muggia et al., 2020; Nelsen et al., 2021; Bordenave et al., 2022). While 29 *Trebouxia* species have been formally described to date based on the combination of morphological traits and genetic diversity (Friedl, 1989a,b; Muggia et al., 2018, 2020; Bordenave et al., 2022), the majority of species-level lineages in this important algal genus lack formal description. Data on their biogeographic and ecological patterns, as well as on physiological traits, are largely missing. Muggia et al. (2020) assembled the most comprehensive taxon sampling for *Trebouxia* and provided a genus-wide, multi-locus phylogenetic hypothesis to use as reference. In their study, the authors confirmed the recognition of four main *Trebouxia* clades—i.e., clade “A” *arboricola*/*gigantea* type, clade “C” *corticola* type, clade “I” *impressa*/*gelatinosa* type, and clade “S” *simplex*/*jamesii* type—within which they further identified some major new species-level lineages (Muggia et al., 2020). More recently, Xu et al. (2020) provisionally segregated some *Trebouxia* algae belonging to clade “S” into a new clade “D,” as these photobionts formed a well-supported monophyletic lineage and were found to specifically associate with the lichen-forming fungus *Cetrariella delisei*. Clade “D” is supported in phylogenetic analyses based on four loci but still lacks a proper morphological characterization (Xu et al., 2020), and some studies still considered it as part of clade “S” (Muggia et al., 2020; Nelsen et al., 2021).

Better characterization of diagnostic traits among distinct *Trebouxia* species-level lineages will be key to creating a robust, integrative taxonomy for this genus. Typical taxonomically diagnostic traits are based on the structure and the extension of the chloroplast lobes, and the distinct arrangement of thylakoid and osmiophilic pyrenoglobules in the pyrenoid (Friedl, 1989a,b; Muggia et al., 2014b,^2018^; Molins et al., 2018a; Bordenave et al., 2022). To perform reliable analyses of these traits, axenic cultures of *Trebouxia* phycobionts are essential. In fact, only in algae grown in standardized *in vitro* conditions is it possible to reliably analyze morphological and physiological traits of putative species-level lineages. Culturing algal cells is also important to correlate the *in vitro* traits with those exhibited in the symbiotic state inside the lichen thalli. Bordenave et al. (2022) recently compiled a new morphological and ultrastructural characterization of 20 *Trebouxia* species-level lineages, reappraising and implementing the classification of *Trebouxia* in accordance with the phylogenetic delimitation provided by Muggia et al. (2020).

The presence of many new phylogenetic clades for *Trebouxia* highlights that species diversity in this genus has been underestimated, with a high proportion of previously unrecognized diversity recently found in undersampled geographic areas (Muggia et al., 2014b; Leavitt et al., 2015). Furthermore, crustose lichens seem to be cradles for new *Trebouxia* species-level lineages, as these lichens usually are more rarely collected and studied with molecular techniques. In the present study, we investigated in more detail the *Trebouxia* species diversity in the two epilithic cosmopolitan lichens *Rhizoplaca melanophthalma* (DC.) agg. Leuckert & Poelt and *Tephromela atra* (Huds.) Hafellner (Muggia et al., 2008, 2014a,b; Leavitt et al., 2013b,Leavitt et al., 2016) collected from high altitudes in rather extreme environments. Both *R. melanophthalma* and *T. atra* represent species complexes, with distinct members within each complex potentially occurring sympatrically (Leavitt et al., 2013b,c, 2016; Muggia et al., 2014a,b). However, throughout the manuscript, the two species groups are referred to by their traditional names. Indeed, previous studies aimed at characterizing the morphological and genetic diversity of their mycobionts and photobionts, respectively, have also highlighted the presence of new, rather specific *Trebouxia* lineages associating with both mycobiont species complexes (Muggia et al., 2008, 2014a,b; Leavitt et al., 2013c,^2016^). While *R. melanophthalma* was found to associate with a relatively narrow range of photobionts (Leavitt et al., 2013a,b), 12 clades of *Trebouxia* were recovered to associate with *T. atra*, five of which (clades I–V in Muggia et al., 2014b) were identified as new, well-supported, distinct lineages.

Here, we aim to assess the potential of unidentified *Trebouxia* species which may represent locally adapted photobionts in rather extreme montane environments worldwide. Taking as reference models the two lichens *R. melanophthalma* and *T. atra*, we will be able to detect whether the recovered chlorobionts represent new *Trebouxia* lineages specific to the peculiar environments where the thalli were collected or if they are part of the already identified photobiont pool with which the two mycobionts associate. We implement an integrative taxonomic approach, combining morphological and ultrastructural data from axenic cultures and thallus sections with genetic data obtained both from culture isolates and the original corresponding lichen thalli. We hypothesize that *R. melanophthalma* and *T. atra* collected at high elevation and in very dry conditions share *Trebouxia* photobionts unique to this type of habitats, while we expect to recover already-identified species-level lineages from samples at lower altitude and less selective environmental conditions. The works of Muggia et al. (2020) and Bordenave et al. (2022) are here used as references for naming the species-level lineages and characterizing their phenotypic traits, respectively.

## Materials and Methods

### Sampling

Two species of epilithic cosmopolitan lichen-forming fungi, i.e., *Rhizoplaca melanophthalma* agg. and *Tephromela atra*, were collected in 43 sites worldwide, the majority of them on different mountain chains, including the Alps in Europe, the Andean Cordillera in South America, and the Rocky Mountains in North America. Most of the sampling sites are located at middle to high altitudes, e.g., above 1,400 m a.s.l., and are characterized by rather extreme oligotrophic environmental conditions. Only two sampling sites from Chile and Tasmania are located below 1,000 m a.s.l., but still in remote mountainous areas (Table 1). At the sampling sites, up to 15 thalli were collected for each population of either species; both species were sometime co-present. A total of 32 populations (67 total thalli) of *R. melanophthalma* agg. and 21 populations (40 total thalli) of *T. atra* were used in this study for both thallus DNA extractions and culture isolations of the photobionts.

**TABLE 1:** Metadata of the collected lichen specimens: species name, sample ID, and geographic origin, including altitude and type of substrate, are reported.

Lichen species	Thallus ID	Altitude (m a.s.l.)	Rock type	Geographic origin
*Rhizoplaca cf. melanophthalma*	L2384	1450	Basaltic boulders	(1) Argentina, prov. Mendoza, dep. Malargue, Laguna de Llancanelo, RP186, 20 km after the crossroad with RN40; S/SW exposed, scattered in dry pampa vegetation, ca. 35°42′50′′S/69°27′18′′W (*L. Muggia*)
*Rhizoplaca cf. melanophthalma*	L2385(*1)			
*Rhizoplaca cf. melanophthalma*	L2388(*2)			
*Rhizoplaca cf. melanophthalma*	L2389(*3)			
*Rhizoplaca cf. melanophthalma*	L2398(*4)	1450	Basaltic/vulcanic rocks	(2) Argentina, prov. Mendoza, dep. Malargue, payunia, 60 km W from Carapacho village and Laguna de Llancanelo, gravel road leading to Puesto Forquera/Payen Matrù, pampa vegetation, on S side of the rocks, ca. 36°12′40′′S/69°11′35′′W (*L. Muggia*)
*Rhizoplaca cf. melanophthalma*	L2400(*5)			
*Rhizoplaca cf. melanophthalma*	L2421(*6)	2000	Basaltic boulders	(3) Argentina, prov. Mendoza, dep. Malargue, El Sosneado valley, Laguna el Sosneado, S/SW exposed, dry pampa vegetation, ca. 34°50′43′′S/69°54′55′′W (*L. Muggia*)
*Rhizoplaca cf. melanophthalma*	L2428			
*Rhizoplaca cf. melanophthalma*	L2452(*7)	3550	Acid big boulders	(4) Argentina, prov. Mendoza, Tunuyan, Cordillera del los Andes (E side), road 94 toward portillo Argentino, camp “Yareta,” 3550 m a.s.l., on acid big boulder, E-S exposed (*L. Muggia*)
*Rhizoplaca cf. melanophthalma*	L2455(*8)			
*Rhizoplaca cf. melanophthalma*	L2460(*42)	3330	Acid rocks	(5) Argentina, prov. Mendoza, Cordillera de los Andes (E side), Las Cuevas, lowest border of Mt. Tolosa glacier, S-W exposed (*L. Muggi*a)
*Rhizoplaca cf. melanophthalma*	L2505	4813	Acid rocks	(6) Argentina, prov. Mendoza, Potrerillo, Cordillera de los Andes (E side), Cordon del Plata Range, Quebrada del Salto, ridge between Cerro El Salto and Cerro Blanco, E-exposed, ca. 32.91376 S/69.40169 W (*L. Muggia*)
*Rhizoplaca cf. melanophthalma*	L2513	5100		(7) Argentina, prov. Catamarca, dep. Fiambalà, Ojo del el Salado, road toward the Ojo del el Salado (*A. E. Armesto*)
*Tephromela atra*	L2545(*9)	600		(8) Chile, Region de Aysén del General Carlos Ibanez del Campo, prov. Capitan Prat, dep. Cochrane, Tamango National Reserve (*J. Orlando and D. Leiva*)
*Tephromela atra*	L2551(*10)			
*Tephromela atra*	L2560(*11)			
*Tephromela atra*	L2561(*12)			
*Rhizoplaca cf. melanophthalma*	L2567(*13)	2080	Siliceous-granitic boulders	(9) Europa, Spain, prov. Madrid, Miraflores del la Sierra, Puerto de la Morquera, summit of Pico Najarra, ca. 40°48′55′′N/3°49′34′′W (*L. Muggia* and *S. Perez-Ortega*)
*Tephromela atra*	L2570(*14)			
*Tephromela atra*	L2571			
*Tephromela atra*	L2583(*15)	1900	Siliceous-granitic boulders	(10) Europe, Spain, prov. Madrid, Miraflores del la Sierra, Puerto de la Morquera, toward Pico Najarra, about 150 m above Puerto de la Morquera, ca. 40°49′22′′N/3°49′49′′W (*L. Muggia* and *S. Perez-Ortega*)
*Rhizoplaca cf. melanophthalma*	L2585(*16)			
*Rhizoplaca cf. melanophthalma*	L2589(*17)			
*Rhizoplaca cf. melanophthalma*	L2593(*18)			
*Tephromela atra*	L2597(*19)	545	Dolorite boulders	(11) Australia, Tasmania, three Thumbs, summit area, 42°36′S/147°52′E, Grid; 570752828/Grid. Sq.: 5728; in dry sclerophyll forest (*G. Kantvilas*)
				
*Tephromela atra*	L2598(*20)			
*Tephromela atra*	L2599(*21)			
*Rhizoplaca cf melanophthalma*	L2635(*22)		Quartzite outcrop	(12) United States, Utah, Utah Co., Rock Canyon, ca. 2 km from trailhead, on exposed quartzite outcrop on north-facing side of canyon; 40.2649, −111.6179 - (Leavitt 19-303)
*Rhizoplaca cf melanophthalma*	L2669(*23)	1665	Sandstone boulders	(13) United States, Utah, Emery County, vic. of Horse Canyon Rest Area along US Highway 6, on sandstone in Pinyon/Juiper woodland: 39.4123, −110.4320
*Rhizoplaca cf melanophthalma*	L2671(*24)			
*Rhizoplaca cf melanophthalma*	L2689(*25)	2020	Wasatch Formation	(14) United States, Utah, Rich Co., southeast of Bear Lake along Highway 30 and west of Sage Creek Junction, on rock in sage-steppe habitat (Leavitt 19–157)
*Rhizoplaca cf melanophthalma*	L2688(*43)			
*Rhizoplaca cf melanophthalma*	L2705(*26)	2490	Sandstone boulder	(15) United States, Utah, Duchesne Co.; Ashley National Forest; South Unit, on Nutter’s Ridge, on sandstone outcrup north-east of exclusure site: 39.9481–110.4292
*Rhizoplaca cf melanophthalma*	L2722(*27)	1845	Basalt/volcanic rocks	(16) United States, Idaho, Owyhee Co. Along Mud Flat Rd, 27.7 miles from Hishway 78. 42.704228–166.3832 (Leavitt 19.233)
*Rhizoplaca cf melanophthalma*	L2723(28*)			
*Rhizoplaca cf melanophthalma*	L2724(*29)			
*Rhizoplaca cf melanophthalma*	L2725(*30)			
*Rhizoplaca cf melanophthalma*	L2732(*31)	2210	Silicic ash flow tuff	(17) United States, Nevada, Nye Co., Humboldt-Toiyabe National Forest, Table Mountain Wilderness Area, near boundary of Table Mountain Wilderness Area, along USFS Road No. 4409b, at Mosquito Creek Trailhead.; 38.80717–116.682
*Rhizoplaca cf melanophthalma*	L2733(*32)			
*Rhizoplaca cf melanophthalma*	L2734(*33)			
*Rhizoplaca cf melanophthalma*	L2735(*34)			
*Rhizoplaca cf. melanophthalma*	L2787(*35)	2700	Acidic rocks	(18) Argentina, prov. Mendoza, road RP52, near to Paramillo, ca. 30 m above the road, ca. 32°30′13′′S/69°03′18′′W (*L. Muggia*)
*Rhizoplaca cf. melanophthalma*	L2796(*36)			
*Rhizoplaca cf. melanophthalma*	L2803(*37)	4300	Acidic rocks	(19) Argentina, prov. Mendoza, dep. Tunuyan, valley toward Portillo Argentino (RN86), summit of Cerro Punta Negra (*L. Muggia*)
*Rhizoplaca cf. melanophthalma*	L2802(*38)			
*Rhizoplaca cf. melanophthalma*	L2824(*39)	3650	Basic granitic rocks	(20) Argentina, prov. Mendoza, dep. Tunuyan, valley toward Portillo Argentino (RN86), ca. 100 height m above the bridge/bifurcation with the road toward Manantiales Valley (*L. Muggia*)
*Rhizoplaca cf. melanophthalma*	L2825			
*Rhizoplaca cf. melanophthalma*	L2826(*40)			
*Rhizoplaca cf. melanophthalma*	L2827(*41)			
*Tephromela atra*	L3272	2200	Siliceous rocks	(21) Italy, Trentino Alto Adige, prov. Trento, Pergine Valsugana, Valley of Mocheni, Mt. Gronlait, 100 height meter below summit, N side of the path, E/N-exposed, ca. 46°05′39 ′′N/11°21′42′′E (*L. Muggia* and *A. Cometto*)
*Tephromela atra*	L3273			
*Tephromela atra*	L3280	2150	Siliceous rocks/cliffs	(22) Italy, Trentino Alto Adige, prov. Trento, Pergine Valsugana, Val dei Mocheni, Passo La Portella, S-exposed, ca. 46°05′38′′N/11°21′57′′E (*L. Muggia and A. Cometto*)
*Rhizoplaca cf melanophthalma*	L3287(*44)	2300	Siliceous rocks	(23) Italy, Trentino Alto Adige, prov. Bolzano, MaziaValley (Matschertal), path to Tartscher Kreuz, boulders in open meadow, W-and S-exposed, ca. 46°41′57′′N/10°35′45′′E (*L. Muggia and A. Cometto*)
*Rhizoplaca cf melanophthalma*	L3293			
*Tephromela atra*	L3314(*45)			
*Tephromela atra*	L3317			
*Rhizoplaca cf melanophthalma*	L3335(*46)	2100	Siliceous rocks	(24) Italy, Trentino Alto Adige, prov. Bolzano, Mazia Valley (Matschertal), path to Tartscher Kreuz, on boulders in open meadow, S-exposed, ca. 46°41′33′′N/10°35′49′′E (*L. Muggia and A. Cometto*)
*Rhizoplaca cf melanophthalma*	L3336(*47)			
*Rhizoplaca cf melanophthalma*	L3340			
*Tephromela atra*	L3352			
*Tephromela atra*	L3358			
*Rhizoplaca cf melanophthalma*	L3365(48*)	2370	Siliceous-granic boulders	(25) Italy, Lombardia, prov. Sondrio, Valmalenco, Chiesa di Valmalenco, at Laghetti di Sassersa, S side of first (lower) lake, S-exposed, ca. 46°16′30′′N/9°48′47′′E (*L. Muggia and A. Cometto*)
*Tephromela atra*	L3396	1650	Siliceous/shists tiles	(26) Italy, Piemonte, prov. Verbania-Cusio-Ossola, Val Vigezzo, Alpe Villasco, on roof tile, N-exposed (*L. Muggia and A. Cometto*)
*Tephromela atra*	L3398			
*Tephromela atra*	L3404(*49)	2300	Granitic boulders	(27) Italy, Aosta Valley, saddle below Mt. Chaligne S/E side, alpine vegetation, ca. 45°46′08′′N/7°14′52′′E (*L. Muggia A. Cometto*)
*Tephromela atra*	L3405			
*Rhizoplaca cf melanophthalma*	L3419(*50)			
*Rhizoplaca cf melanophthalma*	L3422(*51)			
*Rhizoplaca cf melanophthalma*	L3438	2510	Granitic-schist boulders	(28) Italy, Aosta Valley, prov. Aosta, Punta Chaligne, on the saddle N side of the summit, ca. 45°46′16′′N/7°14′06′′E (*L. Muggia* and *A. Cometto*)
*Rhizoplaca cf melanophthalma*	L3440			
*Tephromela atra*	L3470			
*Tephromela atra*	L3471			
*Tephromela atra*	L3472(*53)	1950	Silecous bricks/rocks	(29) Italy, Aosta Valley, prov. Aosta, Gressoney Valley, path to Colle Pinter, Alta Via n. 1, about 100 height meter above Alm Alpenzu, N/W/S-exposed, ca. 45°48′13′′N/7°48′50′′E (*L. Muggia* and *A. Cometto*)
*Tephromela atra*	L3474			
*Rhizoplaca cf melanophthalma*	L3481	2800	Granitic-siliceous cliff	(30) Italy, Aosta Valley, prov. Aosta, Gressoney Valley, Colle Pinter, Alta Via n. 1 (AV1, path n. 6), big cliffs right above the pass, S/W-exposed, 45°49′12′′N/7°47′14′′E (*L. Muggia* and *A. Cometto*)
*Rhizoplaca cf melanophthalma*	L3484			
*Rhizoplaca cf melanophthalma*	L3496	2250	Granitic boulders	(31) Italy, Aosta Valley, prov. Aosta, Gressoney Valley, path to Colle Pinter, Alta Via n. 1 (AV1, path n. 6), before at Alpe Loasche, S-exposed, ca. 45°48′26′′N/7°48′11′′E (*L. Muggia* and *A. Cometto*)
*Rhizoplaca cf melanophthalma*	L3497			
*Tephromela atra*	L3523(*54)	1550	Siliceous rocks/cliffs	(32) Italy, Aosta Valley, prov. Aosta, Gressoney Valley, Alta Via n. 1 (AV1, path n. 6), path from Gressoney to Alpe Alpenzu, S/E-exposed, ca. 45°48′263′′N/7°48′11′′E (*L. Muggia* and *A. Cometto*)
*Tephromela atra*	L3536	1750	Granitic boulders	(33) Italy, Piemonte, prov. Turin, Valley D’ Ala (Lanzo Valley), Ala di Stura, loc. Balme, path n. 228 to Lago Ru, open Larix vegetation on broad bankings, S-exposed (*L. Muggia* and *A. Cometto*)
*Rhizoplaca cf melanophthalma*	L3538(*55)			
*Rhizoplaca cf melanophthalma*	L3540			
*Tephromela atra*	L3555	1500	Granitic rocks	(34) Italy, Piemonte, prov. Turin, Valley D’ Ala (Lanzo Valley), Ala di Stura, loc. Balme, path n. 228 to Lago Ru, at bifurcation with the path to climbing crag “Le Ginevre,” 100 height m above Balme, shadowed, 45°18′11′′N/7°12′56′′E (*L. Muggia* and *A. Cometto*)
*Tephromela atra*	L3559			
*Rhizoplaca cf melanophthalma*	L3564			
*Rhizoplaca cf melanophthalma*	L3572			
*Rhizoplaca cf melanophthalma*	L3576(*56)	1410	Granitic boulders	(35) Italy, Piemonte, prov. Turin, Valley D’ Ala (Lanzo Valley), Ala di Stura, loc. Balme, before entering the village, in front of basketball field, 45°18′13′′N/7°13′23′′E (*L. Muggia* and *A. Cometto*)
*Rhizoplaca cf melanophthalma*	L3577(*57)			
*Rhizoplaca cf melanophthalma*	L3594(*58)	2500	Siliceous rocks/boulders	(36) Italy, Piemonte, prov. Cuneo (Alpi Cozie) Varaita Valley, alpine meadows, main road going up to Colle dell’ Agnello, S-exposed, 44°40′42′′N/6°59′18′′E (*L. Muggia* and *A. Cometto*)
*Rhizoplaca cf melanophthalma*	L3616	2250	Siliceous rocks/boulders	(37) Italy, Piemonte, prov. Cuneo (Alpi Cozie), Val Varaita-Val Maira, Colle di Sampeyre, W of the pass, 44°33′06′′N/7°07′05′′E (*L. Muggia* and *A. Cometto*)
*Rhizoplaca cf melanophthalma*	L3617			
*Tephromela atra*	L3648			
*Rhizoplaca cf melanophthalma*	L3653(*59)	2340	Marmor-siliceous rocks	(38) Italy, Piemonte, prov. Cuneo (Alpi Cozie), Val Maira, Preit, Colle Solegno Blue, rock right above N/E of the pass, S-exposed, 44°26′21′′N/7°01′53′′E (*L. Muggia* and *A. Cometto*)
*Rhizoplaca cf melanophthalma*	L3655(*60)			
*Tephromela atra*	L3681(*61)	1650	Schist-siliceous rocks	(39) Italy, Piemonte, prov. Cuneo (Alpi Cozie), Val Maira, Preit, path to Colle Solegno Blue, shadowed, S-exposed (*L. Muggia* and *A. Cometto*)
*Tephromela atra*	L3696(*62)	2100	Schist-arenaria rocks	(40) Italy, Piemonte, prov. Cuneo (Alpi Marittime), Mt. Ventoso, below the summit, W-exposed, ca. 44°04′56′′N/7°42′58′′E (*L. Muggia* and *A. Cometto*)
*Tephromela atra*	L3697(*63)			
*Tephromela atra*	L3710(*64)	2100	Schistous rocks/cliffs	(41) Italy, Piemonte, prov. Cuneo (Alpi Marittime), Mt. Saccarello, N side below summit, N-exposed, ca. 44°03′45′′N/7°42′43′′E (*L. Muggia* and *A. Cometto*)
*Tephromela atra*	L3720	2150	Schist-arenaria rocks	(42) Italy, Piemonte, prov. Cuneo (Alpi Marittime), Mt. Saccarello, few meters S/E of the summit, S-exposed, ca. 43°03′40′′N/7°42′46′′E (*L. Muggia* and *A. Cometto*)
*Tephromela atra*	L3722(*65)			
*Rhizoplaca cf melanophthalma*	L3724(*66)			
*Rhizoplaca cf melanophthalma*	L3725(*67)			
*Tephromela atra*	L3820	2000	siliceous rocks	(43) Italy, Friuli Venezia Giulia, prov. Udine, loc. Treppo Carnico, Mt. Paularo, on the crest E of summit, E/N-exposed, ca. 46°34′15′′N/13°02′59′′E (*L. Muggia*)
*Tephromela atra*	L3821			

### Culture Isolation of *Trebouxia* Photobionts

The isolation of photobionts was performed by picking the algal cells from different parts of each selected thallus (107 total thalli). We selected three thallus areolas and three lobes, for *T. atra* and *R. melanophthalma* agg., respectively, which were distantly located from each other in the thallus. This procedure aimed at isolating potential intrathalline *Trebouxia* algae diversity, i.e., multiple algal species coexisting within a single lichen thallus. The thallus surface was washed three times by pipetting a sterile solution of 1% Tween-80 in H_2_O. The upper cortex of the thallus was removed with a sterile razor blade, and clumps of algal cells were picked from the algal layer with a sterile needle and directly inoculated on Bold Basal Medium (BBM; Bold, 1949; Bischoff and Bold, 1963) and stored in a growth chamber at 20°C, 20 μmol × photons m^–2^ × s^–1^, with a light/dark cycle of 14/10 h. Once algal colonies grew to a sufficient biomass (about 1-mm-wide colony), they were individually sub-cultivated on BBM and *Trebouxia* medium (TM; Ahmadjian, 1967). In a second sub-cultivation step, part of the colonies were picked for DNA extraction and molecular sequence identification (see below), morphological analysis, and cryostock preservation. The algal cultures are stored both as fresh living strains and as cryostocks at the Department of Life Sciences, University of Trieste.

### Molecular Analyses: DNA Extraction, PCR Amplification, and Sequencing

The total genomic DNA was extracted from both lichen thalli and the isolated photobiont strains following the C-TAB protocol according to Cubero et al. (1999). The nuclear internal transcribed spacer of the ribosomal DNA (ITS rDNA) was amplified using the *Trebouxia*-specific primers ITS1T and ITS4T (Kroken and Taylor, 2000) using the PCR conditions as in Muggia et al. (2014a). The PCR products were purified with Mag-Bind^®^ Total Pure NGS and sequenced by Macrogen Europe, Inc. (Amsterdam, Netherlands) using the forward primer ITS1T. Sequence identity was checked with a BLAST search (Altschul et al., 1990) in the NCBI database and was used to find correspondence between the sequence obtained from the thallus extraction and those obtained from the axenically isolated algal strains coming from the same thallus.

### Phylogenetic Analyses

The phylogenetic analyses of *Trebouxia* photobiont included all the newly obtained ITS sequences, i.e., obtained from the thalli and the axenically isolated photobionts (Supplementary Table 2) and 122 sequences (Supplementary Table 1) selected from the most updated reference dataset of Muggia et al. (2020). These latter represent the four main clades of *Trebouxia* formally recognized so far—clades “A,” “C,” “I,” and “S” (Beck et al., 2002; Leavitt et al., 2015; Muggia et al., 2020). All sequences were aligned firstly in a comprehensive dataset selecting *Asterochloris glomerata*, *A. irregularis*, *Vulcanochloris canariensis*, and *V. symbiotica* as out-groups and including reference sequences of the *Trebouxia* clades “A,” “C,” “I,” and “S” to recognize to which of the four clades the new sequences belonged. Secondly, for each individual *Trebouxia* clade—”A,” “I,” and “S” in which the new sequences were recovered (see Results)—a new multiple sequence alignment (MSA) was prepared. The alignments were prepared using MAFFT v7 (Katoh et al., 2002) with a g-ins-i substitution model and GUIDANCE2 (Sela et al., 2015). The alignments were performed firstly using the MAFFT MSA algorithm with 100 bootstrap replicates (masking columns with confidence scores < 0.95) and secondly manually adjusted in BioEdit v7.2.5 (Hall, 1999).

Maximum likelihood (ML) and Bayesian inference (BI) analyses were run for both the whole *Trebouxia* dataset and the individual clades in the CIPRESS web portal (Miller et al., 2010) using the programs RaxML-v8.2.x (Kozlov et al., 2019) and MrBayes v3.2.7a (Huelsenbeck and Ronquist, 2001), respectively. The ML analysis used the GTRGAMMA substitution model, with 1,000 bootstrap replicates. The BI was carried out by setting two parallel runs with six chains over five million generations, starting with a random tree and sampling every 100th step. We discarded the first 25% of the data as burn-in, and the corresponding posterior probabilities (PPs) were calculated from the remaining trees. The phylogenetic trees were visualized in TreeView v1.6.6 (Page, 1996).

We considered to be species-level lineages those clades recovered as individually monophyletic, fully supported and represented by more than two samples [as originally recognized in Leavitt et al. (2015) and Muggia et al. (2020)].

### Analysis of Geographic Distribution, Altitude, and Intrathalline Co-occurrence of *Trebouxia*

The diversity of *Trebouxia* species-level lineages found in our dataset and their geographic distributions were characterized by basic descriptive statistics. *Trebouxia* species diversity was calculated as the percentage of abundance of each species-level lineage across the geographic areas, arranged according to the continents of origin (Europe, North America, and South America Oceania), and altitudinal ranges (0–1,000, 1,001–1,500, 1,501–2,000, 2,001–2,500, >2,500 m a.s.l.) in which the lichen specimens were collected. *Trebouxia* lineages represented by only one sequence were not included in the analysis. We calculated for each geographic area and altitudinal range the relative abundances as the number of samples belonging to a certain species-level lineage on the total number of sequences obtained for that sampling site and altitudinal range, respectively, and express it as percentage values.

The percentage of co-occurrence of multiple *Trebouxia* species in the same thallus was calculated, considering thalli with and without co-occurrence, for which sequence data from thallus extractions and culture isolates differed or were concordant. Patterns of co-occurrence were also analyzed according the altitudinal ranges described about.

### Morphological Analyses of *Trebouxia* (Light Microscopy and Transmission Electron Microscopy)

The analyses were performed on selected specimens identified in the phylogenetic analyses as *Trebouxia* “A52,” *Trebouxia* “A02,” and *T. vagua* “A04” and “A10” (see “Results” section), because we could obtain cultured strains for these species-level lineages which have not been characterized yet. *Trebouxia vagua* was formally described by Voytsekhovich and Beck (2016), and the morphology of the chloroplast was presented; however, since then, a proper analysis of its ultrastructural traits was lacking.

Light Microscopy was used to study the morphological traits of algal cells grown in cultures (Zeiss Axioscope) using ×400 magnification. Algal cells were picked from the colony and mounted in water, slightly pressing on the cover slide. The samples were photographed with an Axiocam MRc5 (Zeiss) digital camera connected to the microscope, and digital images were documented with the program ThorCam (Axio VS40, Zeiss). TEM was applied to study ultrastructural traits of pyrenoid and chloroplast both on the algae observed within the original thallus and on the corresponding axenically cultured strains. For morphological characterization of both pyrenoid and chloroplast types, we referred to the original classifications of Friedl (1989a,b) for pyrenoid types, that of Škaloud et al. (2015) for the chloroplast types, and the recently compiled revision of pyrenoid and chloroplast types done by Bordenave et al. (2022). The following samples and strains were selected: *Trebouxia* “A52” thalli L2385, 2388, and 2389 and cultured strains L2906, L2908, L2912, and L2918; *Trebouxia* “A02” thalli L2421, L2732, and L2796 and cultured strains L2796, L3202, and L3015; *T. vagua* “A04” cultured strain L2943; and *T. vagua* “A10” cultured strain L2957.

Single-cell isolation cultures were prepared for the cultured strains before TEM examinations. These cultures were grown 21 days on solid BBM at 20°C according to Muggia et al. (2018). After this period, the cells were fixed and dehydrated as described by Molins et al. (2018b). In brief, samples were fixed in 2% Karnovsky fixative for 12 h at 4°C, washed three times for 15 min with 0.01 M PBS (pH 7.4), and postfixed with 2% OsO_4_ in 0.01 M PBS (pH 7.4) for 2 h at room temperature. After being washed in 0.01 M PBS at pH 7.4, the samples were dehydrated at room temperature in a graded series of ethanol starting at 50% and increasing to 70, 95, and 100% for no less than 20–30 min at each step. The samples were embedded in Spurr’s resin according to the manufacturer’s instructions. Ultrathin sections (80 nm) were cut, mounted, and stained with 10% uranyl acetate and 0.1% lead citrate using the “Synaptek Grid-Stick Kit,” as described by Moya et al. (2018). The original lichen thalli were fixed and treated as described for the axenic cultures. The ultrathin sections were observed with a JEOL JEM-1010 (80 kV) electron microscope, equipped with a MegaView III digital camera and “AnalySIS” image acquisition software (SCSIE, University of València).

## Results

### Phylogenetic Analyses of *Trebouxia* From Thallus and Cultured Strains

We successfully isolated 212 *Trebouxia* strains in axenic cultures. These strains came from 94 of the 107 selected thalli (including both *Rhizoplaca melanophthalma* agg. and *Tephromela atra*) from 43 distinct localities. DNA extractions and ITS sequencing were successfully obtained for 96 of 107 thalli and for all the 212 axenically isolated strains (Supplementary Table 2).

**FIGURE 1:**
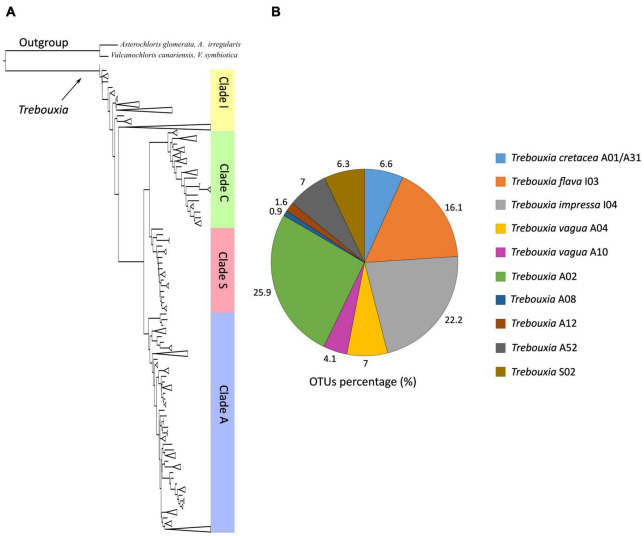
**(A)** Schematic ML and Bayesian phylogenetic hypothesis based on the ITS locus of *Trebouxia* genus including the newly generated sequences. The definition of the major clades follows the multi-locus phylogeny of Muggia et al. (2020). **(B)** Relative abundances of *Trebouxia* species-level lineages recovered in this study.

The phylogeny inferred from ITS sequence data was concordant with the reference phylogeny presented by Muggia et al. (2020) and recognized the four major *Trebouxia* clades (Figure 1A). Bayesian and ML analyses were topologically congruent, and full or high-PP and bootstrap supports were obtained for all lineages. The vast majority of the newly obtained photobiont ITS sequences were placed in clades “A,” “I,” and “S.” Only one sequence was recovered in clade “C” (strain L2759) on a single, unsupported branch, and thus, it was excluded from all subsequent analyses. The individual, clade-specific phylogenies (“A,” “I,” and “S”) were also topologically concordant with the reference trees of Muggia et al. (2020), and the previously delimited species-level lineages were either fully or highly supported (bootstrap values > 80).

**FIGURE 2:**
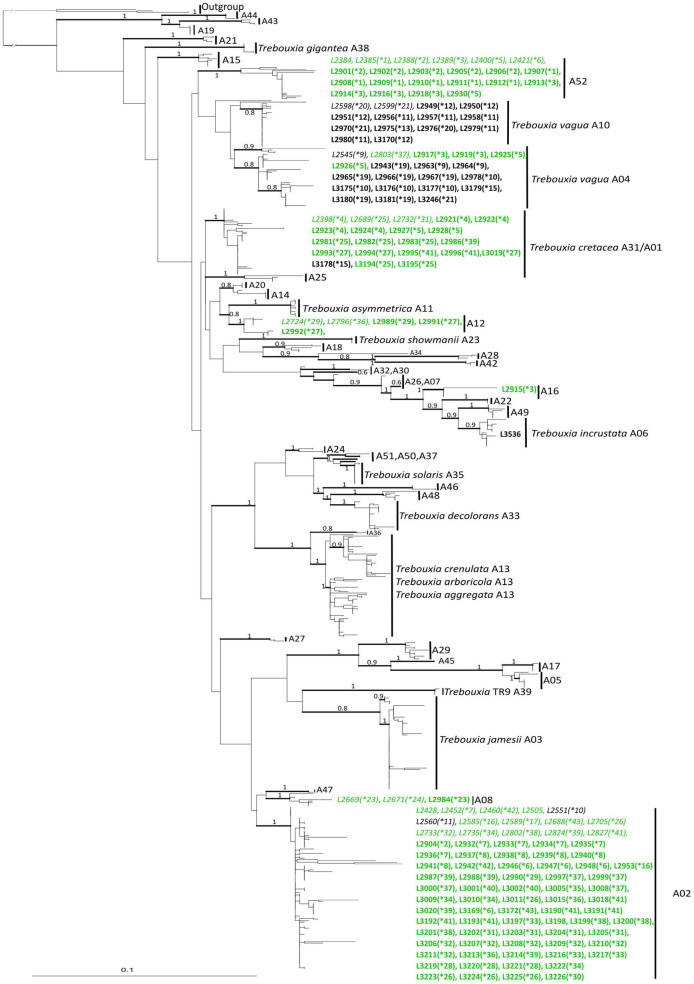
Phylogenetic hypothesis based on the ITS locus of *Trebouxia* Clade “A”: the 50% majority rule consensus tree of the Bayesian analysis is presented; ML bootstrap values higher than 70% are reported with bold branches; Bayesian PP values > 0.8 are reported above branches. DNA extraction numbers of the new *Trebouxia* sequences coming from the original lichen thalli are in *italics*, while those obtained from the cultured strains are in bold. Correspondence between the original lichen thallus and the axenically isolated *Trebouxia* strains is indicated by an asterisk and a number in parenthesis (*1–64; as in Supplementary Table 2). Sequences coming from either lichen species are color coded: green for *Rhizoplaca melanophthalma* and black for *Tephromela atra*.

Most of the *Trebouxia* species-level lineage belong to clade “A,” with 170 sequences representing eight lineages as recognized by Muggia et al. (2020). Among these (Figure 1B), 82 sequences were recognized as *Trebouxia* “A02,” 22 as *T. vagua* “A04,” 21 as *T. cretacea*, 13 as *T. vagua* “A10,” five as *Trebouxia* “A12,” and three as *Trebouxia* “A08,” while *T. incrustata* “A06” and *Trebouxia* “A16” were each represented by a single sequence. Twenty-two additional sequences were recovered within a reciprocally monophyletic clade, closely related to *T. vagua* “A04” and *T. vagua* “A10” and fully supported by both ML and BI analyses. This previously unrecognized putative species-level lineage is here provisionally named *Trebouxia* “A52” (following Leavitt et al., 2015; Muggia et al., 2020). The newly sequenced *Trebouxia* strains in the lineages *Trebouxia* “A52,” *Trebouxia* “A08,” and *Trebouxia* “A12” were derived only from the thalli of *R. melanophthalma*, while those within *T. vagua* “A10” were derived only from *Tephromela atra*. The lineages *T. vagua* “A04,” *Trebouxia* “A02,” and *T. cretacea* group photobionts associated with both species of lichen-forming fungi (Figure 2).

Clade “I” was the second most represented *Trebouxia* clade (Supplementary Figure 1), with 123 sequences coming from both *R. melanophthalma* and *T. atra* specimens. The 123 sequences recovered within clade “I” represented two species-level lineages, i.e., *T. flava* “I03” (54 sequences) and *T. impressa* “I04” (69 sequences).

The 20 sequences recovered in clade “S” (Supplementary Figure 2) come from both *R. melanophthalma* and *T. atra* specimens, and all group into the species-level lineage *Trebouxia* “S02.” Of these, only one sequence belongs to a cultured strain (L4181), while all the other sequences derive from thalli.

### Geographic Diversity of *Trebouxia*

**FIGURE 3:**
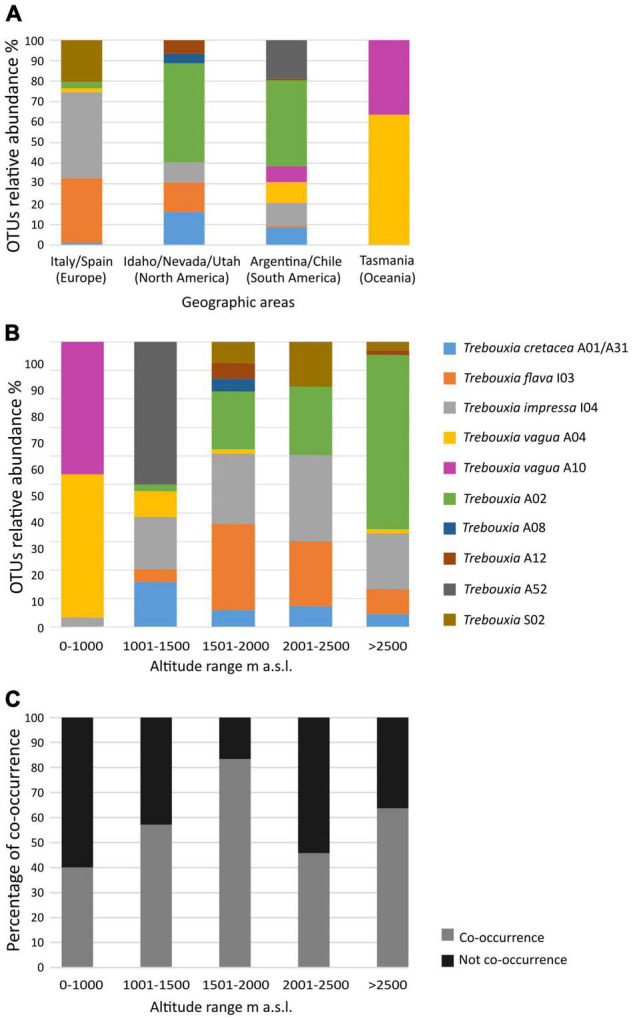
Correlation of relative abundance of *Trebouxia* species-level lineages (OTUs) recovered in thalli of *Rhizoplaca melanophthalma* and *Tephromela atra* **(A)** with the geographic areas according to the continents (Table 1), **(B)** with the altitudinal ranges (m a.s.l.) at which the lichen samples were collected (Table 1). **(C)** Percentage of intrathalline co-occurrence of two or more *Trebouxia* species-level lineages in both *R. melanophthalma* and *T. atra* in relation to the altitudinal range.

In our dataset, *Trebouxia* species-level lineages showed distinct geographic distributions across continents and altitudinal range (Figures 3A,B). In Europe, six *Trebouxia* species-level lineages were found in Italy and Spain, with *T. impressa* “I04,” *T. flava* “I03,” and *Trebouxia* “S02” being the most frequently sampled species. In North America, six different *Trebouxia* species were found, and the most frequently sampled species-level lineages were *Trebouxia* “A02,” *T. cretacea* “A01/A31,” and *T. flava* “I03.” In South America, eight *Trebouxia* species-level lineages were found among Argentina and Chile, with seven found at a single locality in Chile. Here, the most frequent ones were *Trebouxia* “A02” and the new *Trebouxia* “A52.” In Oceania, i.e., from the single locality in Tasmania, we found only the two *Trebouxia* species-level lineages of. *T. vagua* “A04” and “A10.”

*Trebouxia* “A02” and *T. impressa* “I04” were the most broadly distributed species-level lineages and the most abundant in our dataset, representing 25.9 and 22.2% of the new sequences, respectively (Figures 1, 3A,B). Indeed, *Trebouxia* “A02” was most common in thalli from North (Idaho, Nevada, and Utah in the United States) and South America (Argentina and Chile), while only a few samples were found in Europe (Spain). In contrast, *T. impressa* “I04” was more frequent in Europe than in North and South America (Figure 3A). While *Trebouxia* “A02” was mostly amplified and isolated from thalli collected above 1,500 m a.s.l., *T. impressa* “I04” was found also in a few thalli collected in the range 0–1,500 m a.s.l. (Figure 3B).

*Trebouxia flava* “I03” represents 16% of the newly obtained sequences (Figure 1B). This lineage was found frequently in samples from North America (Idaho) and Europe (Italy and Spain), whereas only a few sequences come from South America (Argentina). *Trebouxia flava* “I03” was mostly recovered from thalli collected at mid-high elevation (above 1,000 m a.s.l.) but absent at sites below 1,000 m a.s.l.

*Trebouxia vagua* “A04” and “A10” together represent 11% of the newly obtained sequences. *Trebouxia vagua* “A10” is unique from thalli of both *R. melanophthalma* and *T. atra* coming from the South Hemisphere—as it was found only in Chile and Tasmania (i.e., South America and Oceania)—and collected below 1,000 m a.s.l. *Trebouxia vagua* “A04” instead was also recovered in Argentina and Europe even above 2,000 m a.s.l.

The only previously unsampled species-level lineage *Trebouxia* “A52” represented ∼7% of the newly obtained sequences (Figure 1B) and was recovered only from thalli of *R. melanophthalma* coming from two localities in South America (Argentina) between 1,001 and 1,500 m a.s.l. (Figures 3A,B and Table 1, localities n. 1 and 2).

*Trebouxia* “S02” included about 6.3% of the newly obtained sequences (Figure 1B), and this lineage was present only from samples collected in Europe at sites above 1,500 m a.s.l. (Figures 3A,B).

*Trebouxia cretacea* “A01/A31” represented ∼6% of the newly obtained sequences (Figure 1B) and was recovered from North and South America (Argentina) and Europe (Spain) at sites above 1,000 m a.s.l. In only one locality in Argentina were the samples collected above 2,500 m.

*Trebouxia* “A12” (URa4) represented 1.6% of the newly obtained sequences (Figure 1B) and was found only in thalli from North (Idaho) and South America (Argentina) at sites between 1,500 and 2,000 m a.s.l. and in only one samples above 2,500 m a.s.l.

*Trebouxia* “A08” was the least commonly recovered species-level lineage, representing ∼1% of the new sequences and was found in thalli from North America (Idaho and Utah) at sites between 1,001 and 1,500 m a.s.l. (Figures 1B, 3A,B).

### Correspondence Between Thallus and Axenically Isolated Photobionts

At least two, and up to seven, *Trebouxia* strains isolated from the same original thallus could be sequenced from 48 samples (Supplementary Table 2). Of these, only 12 lichen samples (11 *R. melanophthalma* and one *T. atra*) presented unique correspondence between the thallus photobiont and the axenically isolated photobionts (lichen samples L2385, L2452, L2545, L2689, L2705, L2733, L2735, L2802, L3336, L3365, L3419, and L3594). For the remaining 36 lichen samples (26 of *R. melanophthalma* and 10 of *T. atra*), we found photobiont correspondence, and in addition, we isolated different *Trebouxia* species-level lineages for 11 samples. We found no correspondence at all for 25 samples. The lack of correspondence is due to the following two reasons: (*i*) the isolated strains differed completely from the photobiont amplified from the original thalli (18 samples) and (*ii*) the photobiont sequence could not be obtained from the original thallus (seven samples) and compared with the successfully isolated strains.

In 56 lichen samples, only one cultured strain could be analyzed, and correspondence was found in 10 samples (Supplementary Table 2). Correspondence could not be assessed for the remaining 46 samples because either the thallus sequence or the sequence of the corresponding cultured strains could not be obtained (failure in either PCR amplification or sequencing). Within our dataset, co-occurrence of at least two *Trebouxia* species-level lineages—intrathalline photobiont diversity—was observed in 30.7% of the lichen samples (Figure 3C). The highest percentage of co-occurrence is found in thalli collected in the altitudinal range 1,500–2,000 m a.s.l.

### Morphology and Ultrastructure Analyses of *Trebouxi*a Species-Level Lineages

**FIGURE 4:**
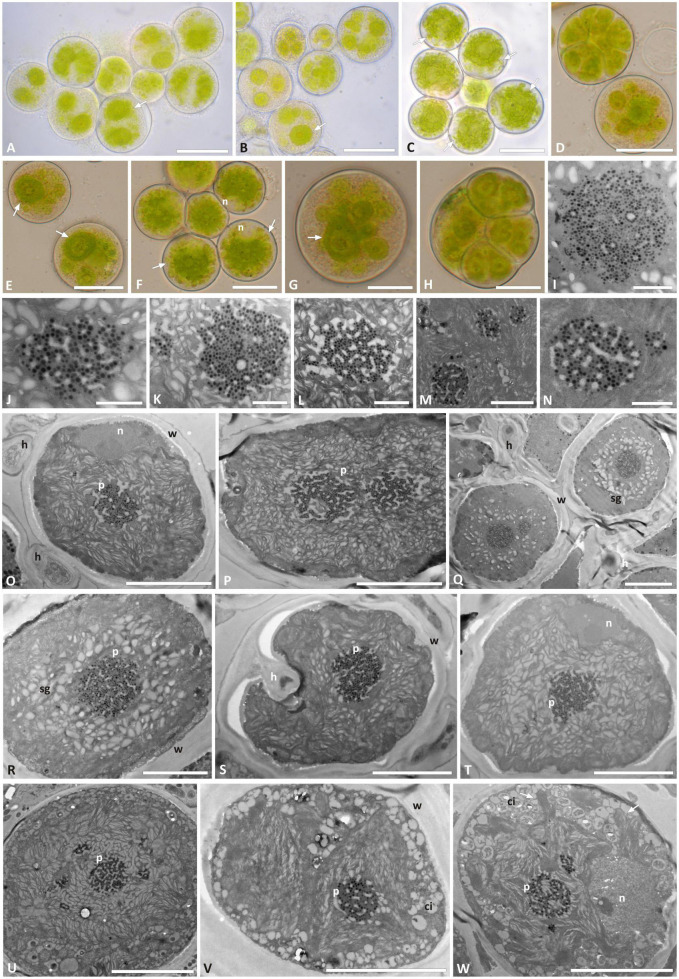
Morphology and pyrenoid ultrastructure of *Trebouxia* “A52” isolated in axenic culture and in the corresponding original thalli. DNA extraction numbers (Table 1 and Supplementary Table 2) identify the samples as follows: **(A–C,U)** culture L2918(*3); **(D–F,W)** culture L2906(*2); **(G,H,M,V)** culture L2903(*2); **(I,Q,R)** from thallus L2388(*2); **(J,K,S,T)** from thallus L2385(*1); **(L,O,P)** from thallus L2389(*3); **(N)** culture L2912(*1). **(A–G)** Cultured algal cells observed by light microscopy: arrows indicate the lobes of the chloroplast (central green body) and the nucleus. **(H)** Asexual autospore cells. **(I–W)** TEM microphotographs of algal cells: **(I–N)** detail of pyrenoid ultrastructure of gigantea type; **(O–S)** algal cells from thallus; **(T–W)** axenically cultured algae. The letters indicate cytoplasmic inclusion (ci), mycobiont hyphae (h), nucleus (n), pyrenoid (p), starch grain (sg), and cell wall (w); multiple pyrenoid bodies are visible in panels **(M,P,Q,U,W)**. Scale bars: **(A–C)** 20 μm; **(D–F,U)** 10 μm; **(G,H,O–T,V,W)** 5 μm; **(M)** 2 μm; **(I–L,N)** 1 μm.

*Trebouxia* “A52” isolates—the previously unsampled species-level lineage—were characterized by regularly coccoid cells of about 15–20 μm diameter (Figures 4A–G) which at maturity form autospores (Figures 4D,H). In LM, the chloroplast occupies almost the whole volume of the cytoplasm forming large lobes, resembling thin branches departing from the central mass (Figures 4A,B,G), which can be attributed to the “deeply lobed” type of chloroplast (Bordenave et al., 2022). TEM analyses of the chloroplast and pyrenoid structures confirmed the observed morphology and revealed the presence of a *gigantea*-type pyrenoid (Figures 4I–W). In a few cells, more than one pyrenoid was detected (Figures 4M,U,W). The nucleus was confined at one side of the cell, likely occupying the biggest invagination in which the chloroplast folds (Figures 4F,O,T,W). Starch grains surrounding the pyrenoid were observed in cells from the thallus (Figures 4Q–S), while cytoplasmic inclusions were observed in the axenically cultured cells (Figures 4I–W).

**FIGURE 5:**
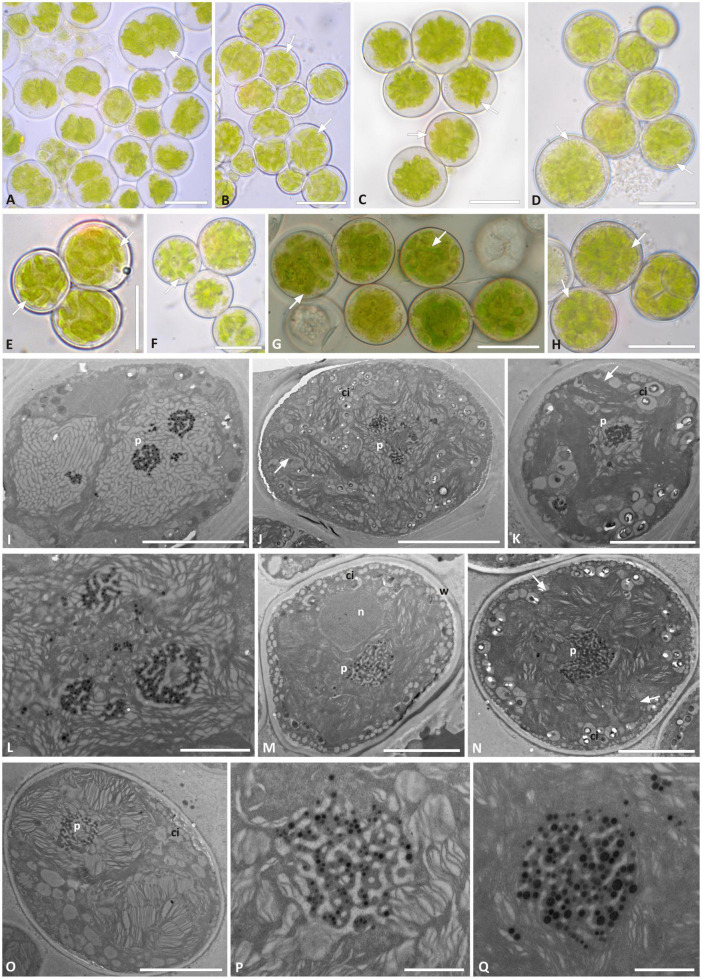
Morphology and pyrenoid ultrastructure of *Trebouxia* “A02” cells isolated in axenic culture. DNA extraction numbers (Table 1 and Supplementary Table 2) identify the samples as follows: **(A–C,E)** L3000; **(D,H–K)** L3015; **(F,M–Q)** L3202; **(G)** L3169. **(A–H)** Algal cells observed by light microscopy; arrows indicate the lobes of the massive central chloroplast. **(I–Q)** TEM microphotographs: (**I**,**J**,**L**) multiple pyrenoid bodies are visible; **(P,Q)** detail of pyrenoid ultrastructure of gigantea type. The letters indicate cytoplasmic inclusion (ci), pyrenoid (p), and cell wall (w). Scale bars: **(A–H,J)** 10 μm; **(I,K,M,N)** 5 μm; **(L)** 2 μm; **(P,Q)** 1 μm.

*Trebouxia* “A02” presents regularly coccoid cells of about 10–15 μm diameter in culture. The chloroplast occupied almost the whole volume of the cytoplasm and forms shallowly elongated lobes (Figures 5A–H), resembling the “shallowly lobed” type of chloroplast (Bordenave et al., 2022). Also, in this taxon, the nucleus was confined at one side of the cell, occupying the biggest invagination of the chloroplast. Similar to *Trebouxia* “A52,” *Trebouxia* “A02” cultured strains also had gigantea-type pyrenoids (Figures 5I–Q), and in some cells, multiple pyrenoids were counted (Figures 5I,L). The ultrastructure of the chloroplast from the original lichen thalli could not be studied for *Trebouxia* “A02” because from the corresponding thalli (L2732 and L2796), from which the analyzed cultured strains (L3202 and L3015) were isolated, sequences belonging to *T. cretacea* and *Trebouxia* “A52” were obtained. Both *T. cretacea* and *Trebouxia* “A52” have a *gigantea*-type pyrenoid; therefore, we could not assess whether the intrathalline algal cells observed at TEM belong to any of the three taxa potentially co-existing within the thalli. Furthermore, the thallus L2796 for which *Trebouxia* “A02” (L3015) and *T. impressa* were isolated (Supplementary Table 2) revealed a thallus sequence belonging to *Trebouxia* “A12.” The TEM analysis performed on this thallus evidenced the presence of cells with two pyrenoid types, i.e., *gigantea* type and *impressa* type (Supplementary Figure 3).

**FIGURE 6:**
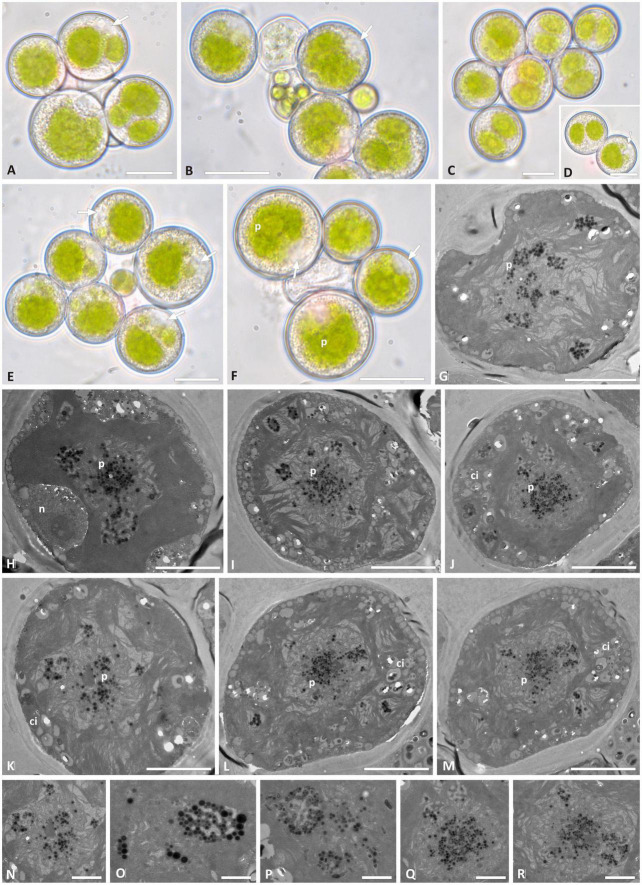
Morphology and pyrenoid ultrastructure of the *Trebouxia vagua* “A04” axenically cultured strain L2943 (Supplementary Table 2). **(A–F)** Algal cells observed by light microscopy; arrows indicate the lobes of the chloroplast (central green body) and the nucleus. **(G–R)** TEM microphotographs of algal cells: **(N–R)** detail of pyrenoid ultrastructure. The letters indicate cytoplasmic inclusion (Ci), nucleus (n), and pyrenoid (p). Scale bars: **(A–F)** 10 μm; **(G–M)** 5 μm; **(N–R)** 2 μm.

*Trebouxia vagua* “A04” presents regularly coccoid cells of about 15–20 μm diameter in culture. The chloroplast occupied most of the volume of the cell and was surrounded by cytoplasmic inclusions that made it hard to distinguish the lobes in LM (Figures 6A–F). The nucleus was confined at one side of the cell, occupying the biggest invagination of the chloroplast (Figures 6A,B,E,F,H). *Trebouxia vagua* “A04” pyrenoids did not resemble any of the so far identified types in *Trebouxia*. The pyrenoid was rather extended in the central part of the chloroplast, and thylakoid lamellae were randomly arranged in it, sometimes even undistinguishable. Pyrenoglobules were grouped in clumps, without a regular arrangement (Figures 6G–R). The intrathalline *T. vagua* “A04” (thallus L2597) could not be compared with the cultured one (L2942; Supplementary Table 2), as the corresponding thallus sequence could not be obtained.

**FIGURE 7:**
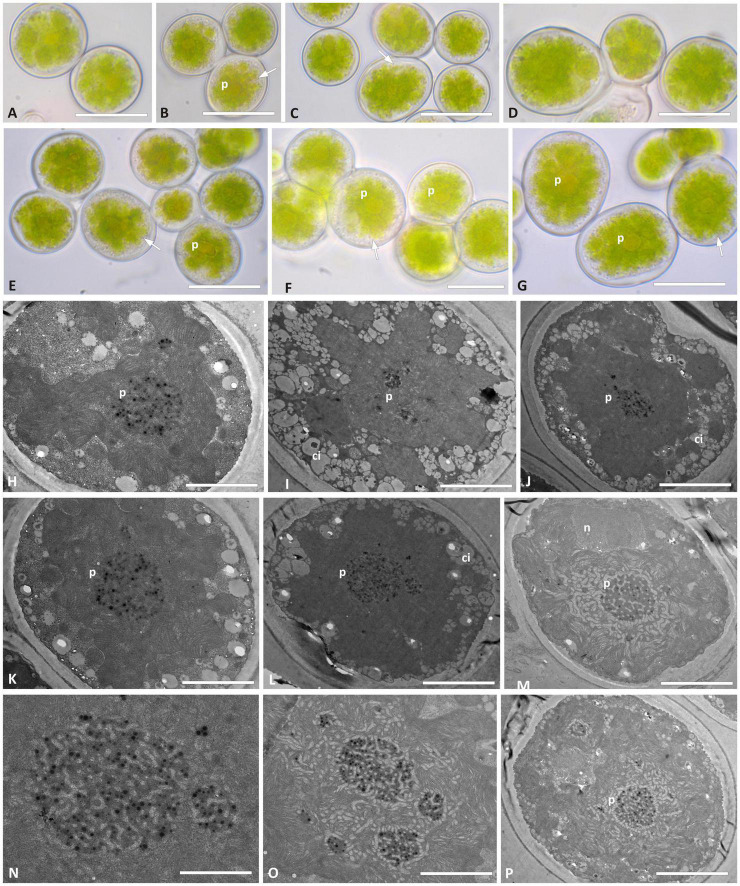
Morphology and pyrenoid ultrastructure of the *Trebouxia vagua* “A10” axenically cultured strain L2957. **(A–G)** Algal cells observed by light microscopy; arrows indicate the lobes of the chloroplast (central green body). **(H–P)** TEM microphotographs; **(N,O)** detail of pyrenoid ultrastructure gigantea type. The letters indicate cytoplasmic inclusion (ci), nucleus (n), and pyrenoid (p). Scale bar: **(E,G)** 20 μm; **(A–D,F)** 15 μm; **(H–J,M,P)** 5 μm; **(K,L)** 2 μm; **(N,O)** 1 μm.

*Trebouxia vagua* “A10” in culture presented coccoid cells of ∼15–20 μm diameter, while bigger cells were roundish to ellipsoid and measured up to 25 μm on the longer axis (Figures 7A–G). The chloroplast massively occupied the cell volume, and its lobe morphology resembled either the curly or the thin-lobed types (*sensu* Bordenave et al., 2022). Abundant granular cytoplasmatic inclusion (Figures 7H–K) prevented the clear distinction of the lobes. *T. vagua* “A10” showed a gigantea-type pyrenoid in culture (Figures 7L–P). Intrathalline cells could not be analyzed in TEM as the thallus sequence (L2560) revealed the presence of *Trebouxia* “A02.”

## Discussion

### Phylogenetic and Ecological Species Diversity of *Trebouxia*

This study explored the diversity of *Trebouxia* photobionts associated with two lichen-forming fungi—*Rhizoplaca melanophthalma* and *Tephromela atra*—originating from environments in remote, exposed areas, at alpine altitudes. There, the lichens thrive under rather extreme ecological conditions and may potentially associate with diverse, ecologically adapted *Trebouxia* species. Our sampling extended almost over the entire distributional range of the two lichen species worldwide and allowed us to elucidate novel insight into ecological distributions of *Trebouxia* species in undersampled regions. For this study, lichen thalli were collected mostly in mountain areas over 2,000 m a.s.l.; five localities were above 3,000 m a.s.l. and up to 5,100 m a.s.l. The environmental extremes that characterize all the sampling localities are draught, extremely low and high temperatures, long snow cover over the year, high levels of UV radiation, and oligotrophic conditions.

Across our global sampling, a total of 10 *Trebouxia* candidate species (species-level lineages) were found to associate with either mycobiont—*R. melanophthalma* or *T. atra*. The greatest diversity of *Trebouxia* species-level lineages was recovered in the altitudinal range 1,000–2,500 m a.s.l. While we found relatively high diversity of *Trebouxia* species and diverse associations of these with the two mycobionts, only a single, previously unsampled species-level lineages was identified—*Trebouxia* “A52.” The nine previously recognized candidate species included *T. vagua* “A04,” *T. cretacea* “A01/A31,” *Trebouxia* “A12,” *Trebouxia* “A08,” *Trebouxia* “A02,” *Trebouxia* “A16,” *T. impressa* “I04,” *T. flava* “I03,” and *Trebouxia* “S02” (Muggia et al., 2020). However, our data reveal that among the four *Trebouxia* main clades, candidate species within clade “A” were most commonly found in *R. melanophthalma* and *T. atra* thalli, while only two species-level lineages of clade “I” and one of clade “S” were identified. Although our molecular results are based on the analysis of the ITS locus alone, we are confident of the correct species-level lineage identification, as topological concordance is found between the phylogenies presented here and the previously published reference studies (Pérez-Ortega et al., 2012; Muggia et al., 2014b,^2020^; Nyati et al., 2014; Leavitt et al., 2015; Voytsekhovich and Beck, 2016; Molins et al., 2018a).

Four of the identified species-level lineages in *Trebouxia* clade “A”—*Trebouxia* “A08,” *Trebouxia* “A12,” *Trebouxia* “A16,” and *Trebouxia* “A52”—were recovered exclusively as partners of *R. melanophthalma*. *Trebouxia* “A08” together with *Trebouxia* “A02” and *T. cretacea* is an already-known photobiont of *R. melanophthalma* based on samples collected in North America and Asia (Leavitt et al., 2015, 2016). In this study, *Trebouxia* “A02,” in particular, has been found as the most frequently associated photobiont in *R. melanophthalma* thalli in our sampling and was recovered across 14 localities, three of these above 3,000 m a.s.l. and two above 4,300 m a.s.l. Only two sequences of *Trebouxia* “A02” were obtained from *T. atra* thalli collected in Patagonia (Chile) at low elevation but in a rather extreme environment. Interestingly, *Trebouxia* “A02” was originally reported from Antarctic lichens by Pérez-Ortega et al. (2012)—and it was equivalent to the species *Trebouxia* “sp. URa2” contemporaneously recognized by Ruprecht et al. (2012). Recently, its dominance in Antarctic lichens (including a *Rhizoplaca* species) from the polar deserts of the McMurdo Dry Valleys has been confirmed by Wagner et al. (2020). Furthermore, Ruprecht et al. (2020) reported *Trebouxia* “A02” also as a very common photobiont in lecideoid lichen species collected on a global scale, including thalli from Patagonia. These previous studies and our present results confirm the widespread distribution of this photobiont, suggesting an adaptation to dry, cold, and oligotrophic environments. *Trebouxia* “A02” has been reported in a total of 14 genera of saxicolous lichens [*Austrolecia*, *Buellia*, *Carbonea*, *Huea*, *Lecanora*, *Lecidea*, *Lecidella*, *Rhizoplaca*, as in Wagner et al. (2020); *Acarospora*, *Caloplaca*, *Polysporina*, *Sarcogyne*, and *Umbilicaria* as in Pérez-Ortega et al. (2012); and *Tephromela*, this study]. Herewith, we succeeded in isolating and maintaining a few strains (isolated from thalli collected at different localities) of *Trebouxia* “A02” in culture and in characterizing its cell morphology and ultrastructure. Nevertheless, further analyses need to be accomplished to fully characterize the physiological traits of this photobiont and formally describe it as a new species.

Our results revealed that members of the *R. melanophthalma* agg. also associate with *T. vagua* “A04,” *Trebouxia* “A16,” *Trebouxia* “A12,” *Trebouxia* “A52,” and *T. flava* “I03.” In particular, the newly recovered species-level lineage *Trebouxia* “A52” was isolated and directly amplified from thalli collected in only three, very dry localities in Argentina (localities n. 1, 2, 3) on basaltic/volcanic rocks. While *R. melanophthalma* represents a species complex comprising multiple, distinct mycobiont species-level lineages (Leavitt et al., 2013a), previous studies suggest that these mycobionts share similar photobiont pools (Leavitt et al., 2016). *Trebouxia cretacea* was described from the Crimean Peninsula (Voytsekhovich and Beck, 2016) and was subsequently found to coexist in thalli of Mediterranean lichen species, such as *Buellia zoharyi* and *Ramalina farinacea* (Molins et al., 2020, 2021; Moya et al., 2020, 2021) and to be widespread in other lichens (Moya et al., 2020). Here, *T. cretacea* was detected in thalli of *R. melanophthalma* from North and South America collected in rather dry conditions.

Six species-level lineages of *Trebouxia* were found in association with the mycobiont *T. atra* (*T. vagua* “A04,” *T. vagua* “A10,” *Trebouxia* “A02,” *T. flava* “I03,” *T. impressa* “I04,” and *Trebouxia* “S02”). We highlight here that *T. atra* associates with a more diverse range of *Trebouxia* species than previously reported (Muggia et al., 2008, 2010, 2014b). Interestingly, in the present study, *T. jamesii* “A03” was never found in association with *T. atra* thalli, whereas Muggia et al. (2010, 2014b) reported this photobiont as extremely common in thalli from Europe and from the northern hemisphere in general. Instead, *T. vagua* “A04” and “A10,” *T. impressa* “I04,” and *Trebouxia* “S02” were the most recovered photobionts associated with the *T. atra* mycobiont. *Trebouxia vagua* “A10” was the one uniquely associated to it. *Trebouxia vagua* “A04” and “A10” were mainly isolated from thalli growing on siliceous/granitic rocks in south Chile (Patagonia; locality n. 8) and Tasmania (locality n. 11), where only populations of *T. atra* were present.

Within *Trebouxia* clade “S,” *Trebouxia* “S02” was the only species-level lineage detected, and it was mainly amplified from the thalli of *T. atra*, while only two sequences were derived from *R. melanophthalma*. Of these, only two thalli came from a mountain massif in Spain, while all the other samples were collected on the Italian Alps. *Trebouxia* belonging to clade “S” and more specifically to the *T. simplex* and *Trebouxia* “S02” lineages have already been documented in Alpine lichens (Blaha et al., 2006; Garrido-Benavent et al., 2020) and have been previously reported for *T. atra* as well (Muggia et al., 2010, 2014a). Although not restricted to the Alps, both *T. simplex* and *Trebouxia* “S02” have been reported from lichen symbioses from cold and rather humid areas, and our survey seems to confirm once again their ecological preferences.

The phylogenetic species diversity of *Trebouxia* detected here corresponds with the evolutionary pathways traversed by its four major clades to occupy varied climatic and vegetative regimes, as recently described by Nelsen et al. (2021). In fact, clade “C” was the first lineage to expand into a regime whose extant members occupy hot and wet climates in partially or exclusively forested habitats (Nelsen et al., 2021). This is the clade that was not found in our sampling, as *R. melanophthalma* and *T. atra* do not commonly occur in wet, forested localities. Instead, for these mycobionts, we showed the greatest *Trebouxia* diversity in clade “A,” which is the clade that originally, exclusively or partially, occupied forested habitats and subsequently extended to occupy regimes characterized by cooler and drier habitats (Nelsen et al., 2021). In a similar way, clade “S” likely occupied originally forested habitats and later expanded into habitats with cooler and drier climates and finally diversified in non-forested habitats (Nelsen et al., 2021). Such habitat descriptions correspond well with the characteristics of the localities visited in this study, the majority of which are dry, cold, wind exposed, and not forested. Finally, Nelsen et al. (2021) inferred clade “I” to have partially or extensively relied on forested habitats, and only isolated lineages would have undergone a transition into regimes whose modern members occupy warmer, as well as cooler and drier, habitats. This also explains congruently why we recovered the two species-level lineages *Trebouxia flava* and *T. impressa* in the dry and cool localities (in Utah, Argentina, and the mountain massif in central Spain). Furthermore, *T. impressa*, similar to that we observed for *Trebouxia* “A02,” is widespread over an altitudinal range above 1,000 m a.s.l. (being reported also for thalli collected above 4,000 m a.s.l.) and was found in the Antarctic region by Ruprecht et al. (2012, 2020).

### Intrathalline Diversity of *Trebouxia* and Morphoanatomical Species Characterization

Co-occurrence of *Trebouxia* photobionts within the same lichen thallus has been recognized to be a common phenomenon for many years (Muggia et al., 2008; Casano et al., 2011; Català et al., 2016; Moya et al., 2017). It was speculated that multiple photobionts may serve in the lichen symbiosis as strategic, additional partners to cope with changeable environmental conditions and to help the mycobiont widen its ecological distribution (Casano et al., 2011). However, this has not been demonstrated on a wide scale. Co-occurring *Trebouxia* photobionts, in addition to a biologically relevant photobiont (Paul et al., 2018), have been discovered worldwide and in diverse lichen symbioses, e.g., lichen thalli of distantly related groups and with different growth forms. However, to date, photobiont co-occurrence does not seem to follow any specific patterns. In the present work, we detected photobiont co-occurrence in 30 of 104 thalli analyzed, and most of these were thalli collected above 1,000 m a.s.l., which leads to speculation about a potential low photobiont selectivity by the two mycobionts, which would allow them to spread over a broad altitudinal range, expanding toward higher elevations.

While more recent analyses highlighted photobiont co-occurrence using DNA metabarcoding data (Moya et al., 2017, 2020, 2021; Paul et al., 2018; Molins et al., 2020; Smith et al., 2020), we implemented here the more traditional but still reliable comparison between the axenically isolated *Trebouxia* strains and the *Trebouxia* sequence obtained from direct amplification from thallus and Sanger sequencing. This approach proved useful in the previous study of Muggia et al. (2014b), when photobiont co-occurrence in *T. atra* was originally reported. In the present study, we provide the first compelling evidence of photobiont co-occurrence in the lichen *R. melanophthalma*. Among the analyzed samples for either lichen species, we detected up to three *Trebouxia* species-level lineages, one being detected from the thallus sequences and the other two corresponding to two different isolated strains from the same thallus.

The multiplicity of algal partners inside a thallus may be interpreted in the light of different symbiotic strategies and flexibility of the symbionts. Indeed, the potential of a lichen mycobiont to host multiple intrathalline photobionts (Casano et al., 2011) led to the possibility to build the best habitat-adapted symbiosis (Rodriguez et al., 2008). This would allow also for photobiont demographic variation within the thallus to ideally match the ecological conditions in which the thallus develops. Under this view, the recognition signaling between the symbionts must be less restricted, likely helping the lichens to colonize different substrates under a wider range of changing conditions, such as those that are found at higher elevation.

Furthermore, in this study, we also show that a detailed analysis of pyrenoid ultrastructure is key in assessing the multiplicity of *Trebouxia* photobionts within thalli. Indeed, as shown for the sample *R. melanophthalma* L2796, the TEM analyses performed on its thallus evidenced the presence of *Trebouxia* cells with two pyrenoid types, i.e., *gigantea* type and *impressa* type. The sequence obtained from thallus L2796 identified the taxon as *Trebouxia* “A12,” while the cultured strains were identified as *Trebouxia* “A02” and *T. impressa* (Supplementary Table 2). The *impressa*-type pyrenoid likely corresponds to cells of *T. impressa* seen in the thallus, but cells with *gigantea*-type pyrenoid may correspond either to the cultured *Trebouxia* “A02” or to the thallus-sequenced *Trebouxia* “A12.” Our TEM analyses conducted on axenically isolated *Trebouxia* “A02” confirm that it has the gigantea-type pyrenoid. However, we cannot exclude that *Trebouxia* “A12” also bears the same gigantea-type pyrenoid, as we could not isolate this photobiont in culture.

Cell morphology and ultrastructure characterization emerges once more as a decisive approach for the comprehensive identification of *Trebouxia* species. Here, we succeeded in isolating and morphologically characterizing two strains, *Trebouxia* “A02” and *Trebouxia* “A52,” which are well-supported, monophyletic species-level lineages in *Trebouxia* clade A and will merit formal species description. However, we refrain to proceed with species description at this stage as further data from still-ongoing research (Medeiros et al., 2021 and future manuscript in preparation) will be merged to obtain a consistent and comprehensive representation of these new taxa. Furthermore, morphology and ultrastructure analyses of the species-level lineage *Trebouxia vagua* “A04” could not distinguish a lobe pattern (partially caused by the presence of abundant cytoplasmic inclusions) but alternatively highlighted a peculiar pyrenoid type, which does not match any previously recognized pyrenoid types in *Trebouxia* (Friedl, 1989a,b; Bordenave et al., 2022). This preliminary observation deserves further inspections of additional strains. Whether the peculiar ultrastructure of *T. vagua* “A04” will be confirmed, this species-level lineage merits further taxonomical consideration. Indeed, the closely related sister lineage *T. vagua* “A10” has been characterized for having a chloroplast with lobe morphology resembling either the curly or the thin-lobed types and a clear gigantea-type pyrenoid.

The presence of multiple trebouxioid taxa within the lichen thalli merits additional consideration, particularly regarding common technical problems related to the formation of chimeric sequences. Indeed, we are aware that the presence of mixed DNA from several different *Trebouxia* taxa within the lichen thalli and/or even in the isolated cultures analyzed could generate a mixed template of the ITS genetic fragments. This could potentially lead to the formation of chimeric sequences that are subsequently amplified in PCR and generate bias in the interpretation of the results. Such troubles could be overcome by cloning or high-throughput sequencing (HTS) approaches, which would detect with a higher accuracy the different ITS sequences. However, this kind of approach was not contemplated in the present study as it will be presented subsequently in a broader context in a future contribution.

In conclusion, the integrative taxonomic approach that has been applied here shed light on the diversity of *Trebouxia* in remote areas characterized by ecological extremes, further strengthening the importance of performing detailed studies on this ecologically adapted and widespread lichen photobiont genus.

## Data Availability Statement

The data presented in the study are deposited in the GenBank repository, accession number OM275483 – OM275791. The original contributions presented in the study are incluted in the article/Supplementary Material, further questions can be directed to the corresponding author.

## Author Contributions

LM, SL, and MG designed the study. LM, AC, and SL performed the sampling. RD and AC performed the culture isolation. RD performed the molecular analyses. PM and EB performed the microscopy analyses. LM, RD, SL, and MT wrote the manuscript. All authors contributed to the article and approved the submitted version.

## Conflict of Interest

The authors declare that the research was conducted in the absence of any commercial or financial relationships that could be construed as a potential conflict of interest.

## Publisher’s Note

All claims expressed in this article are solely those of the authors and do not necessarily represent those of their affiliated organizations, or those of the publisher, the editors and the reviewers. Any product that may be evaluated in this article, or claim that may be made by its manufacturer, is not guaranteed or endorsed by the publisher.

## Supplementary Material

Supplementary Figure 1 Phylogenetic hypothesis based on the ITS locus of *Trebouxia* Clade “I”: the 50% majority rule consensus tree of the Bayesian analysis is presented; ML bootstrap values higher than 70% are reported with bold branches; Bayesian PP values > 0.8 are reported above branches. DNA extraction numbers of the new *Trebouxia* sequences coming from the original lichen thalli are in *italics*, while those obtained from the cultured strains are in bold. Correspondence between the original lichen thallus and the axenically isolated *Trebouxia* strains is indicated by an asterisk and a number in parenthesis (*1–64; as in Supplementary Table 2). Sequences coming from either lichen species are color coded: green for *Rhizoplaca melanophthalma* and black for *Tephromela atra*. Polyphyletic species-level lineage I01 is identified according to Muggia et al. (2020).

Supplementary Figure 2 Phylogenetic hypothesis based on the ITS locus of *Trebouxia* Clade “S”: the 50% majority rule consensus tree of the Bayesian analysis is presented; ML bootstrap values higher than 70% are reported with bold branches, and Bayesian PP values > 0.8 are reported above branches. DNA extraction numbers of the new *Trebouxia* sequences coming from the original lichen thalli are in *italics*, while those obtained from the cultured strains are in bold. Correspondence between the original lichen thallus and the corresponding axenically isolated *Trebouxia* strains is indicated by an asterisk and a number in parenthesis (*1–64; as in Supplementary Table 2). Sequences coming from either lichen species are color coded: green for *Rhizoplaca melanophthalma* and black for *Tephromela atra*. Polyphyletic species-level lineage S02 is identified according to Muggia et al. (2020).

Supplementary Table 1 *Trebouxia* sequences retrieved from GenBank and included in the phylogenetic analyses of Figure 1 and Supplementary Figures 1, 2 are reported with their OTU ID (according to Muggia et al., 2020) and their NCBI accessions.

Supplementary Table 2 NCBI accession numbers are reported for the ITS sequences of *Trebouxia* species-level lineage identified directly from thallus sequencing (thallus ID) and from axenically isolated cultured strains (culture isolates ID). TSB herbarium number is reported for the original lichen samples.
