# Supplementary figures and images for: Photobiont Diversity in Lichen Symbioses From Extreme Environments
Source: Front Microbiol. 2022 Mar 29;13:809804. doi: 10.3389/fmicb.2022.809804 (PMC9002315; doi:10.3389/fmicb.2022.809804)

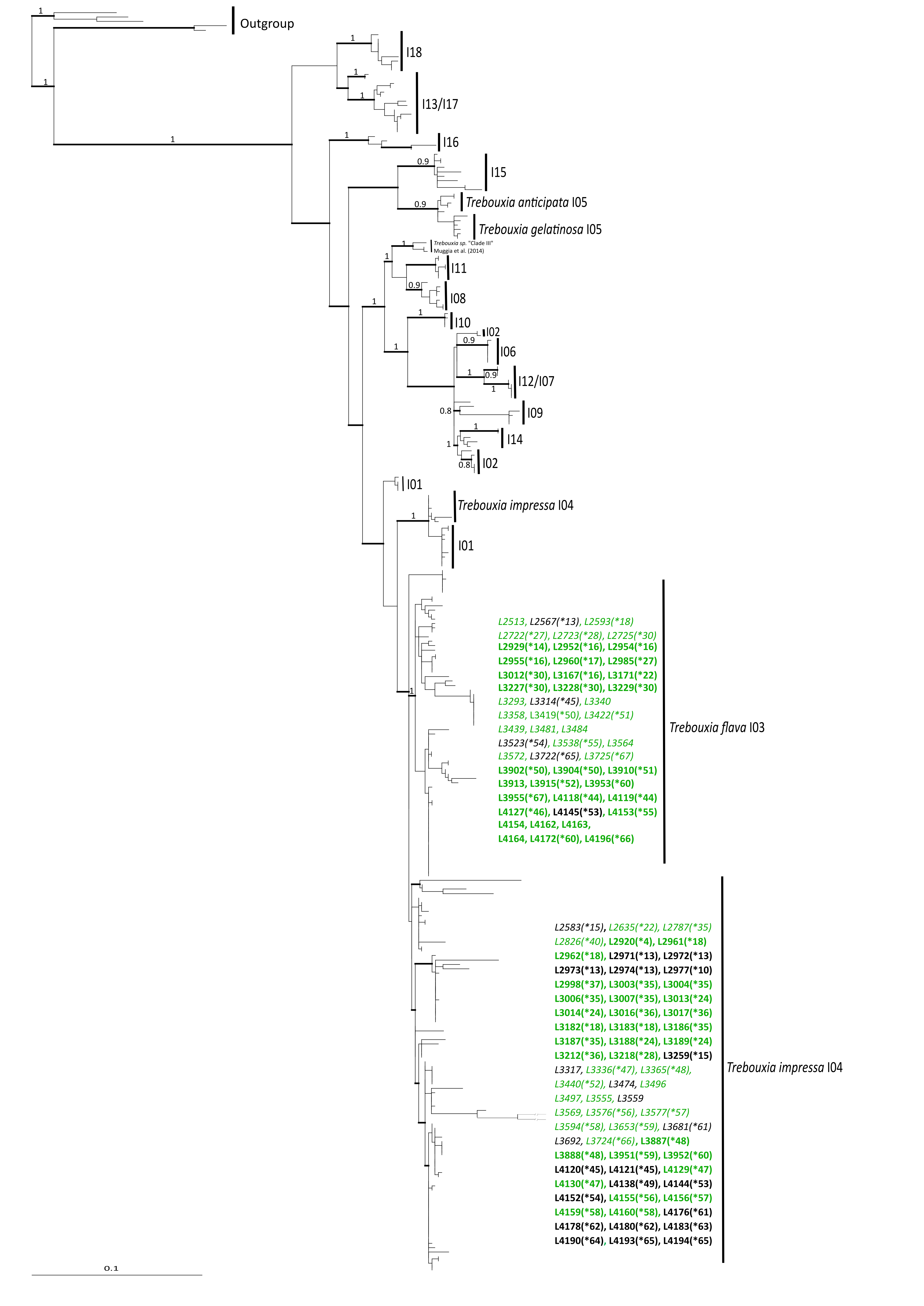

Supplement: Supplementary Figure 1 — Phylogenetic hypothesis based on the ITS locus of Trebouxia Clade “I”: the 50% majority rule consensus tree of the Bayesian analysis is presented; ML bootstrap values higher than 70% are reported with bold branches; Bayesian PP values > 0.8 are reported above branches. DNA extraction numbers of the new Trebouxia sequences coming from the original lichen thalli are in italics, while those obtained from the cultured strains are in bold. Correspondence between the original lichen thallus and the axenically isolated Trebouxia strains is indicated by an asterisk and a number in parenthesis (*1–64; as in Supplementary Table 2). Sequences coming from either lichen species are color coded: green for Rhizoplaca melanophthalma and black for Tephromela atra. Polyphyletic species-level lineage I01 is identified according to Muggia et al. (2020). [file Image_1.PNG]

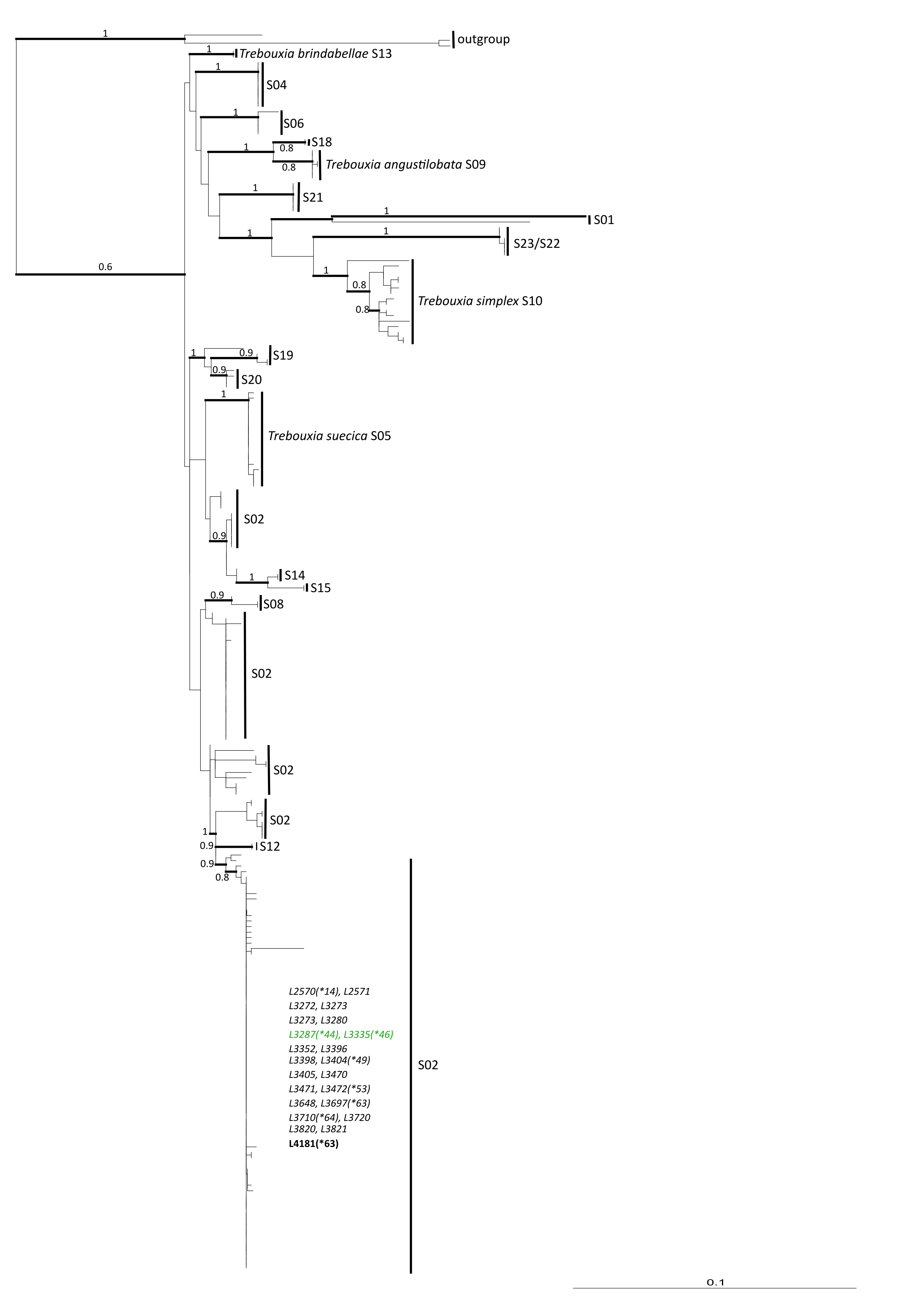

Supplement: Supplementary Figure 2 — Phylogenetic hypothesis based on the ITS locus of Trebouxia Clade “S”: the 50% majority rule consensus tree of the Bayesian analysis is presented; ML bootstrap values higher than 70% are reported with bold branches, and Bayesian PP values > 0.8 are reported above branches. DNA extraction numbers of the new Trebouxia sequences coming from the original lichen thalli are in italics, while those obtained from the cultured strains are in bold. Correspondence between the original lichen thallus and the corresponding axenically isolated Trebouxia strains is indicated by an asterisk and a number in parenthesis (*1–64; as in Supplementary Table 2). Sequences coming from either lichen species are color coded: green for Rhizoplaca melanophthalma and black for Tephromela atra. Polyphyletic species-level lineage S02 is identified according to Muggia et al. (2020). [file Image_2.PNG]
