# Supplementary material for: Photobiont Diversity in Lichen Symbioses From Extreme Environments
Source: Front Microbiol. 2022 Mar 29;13:809804. doi: 10.3389/fmicb.2022.809804 (PMC9002315; doi:10.3389/fmicb.2022.809804)
Supplement: Supplementary Table 1 — Trebouxia sequences retrieved from GenBank and included in the phylogenetic analyses of Figure 1 and Supplementary Figures 1, 2 are reported with their OTU ID (according to Muggia et al., 2020) and their NCBI accessions. [file Data_Sheet_1.DOCX]

| **Specimen Label** | **OTU** | **Study** | **ITS accession** |
| --- | --- | --- | --- |
| *Trebouxia sp* OTU 14 | A14 | Leavitt et al. 2015 | KR913247 |
| Trebouxia asymmetrica | A11 | Leavitt et al. 2015 | KR913196 |
| *Trebouxia australis* | S02 | Bačkor et al. 2010 | FM945345 |
| *Trebouxia australis* | S02 | Leavitt et al. 2015 | KR914198 |
| *Trebouxia australis* | S02 | Fernandez-Mendoza 2011 | GQ375345 |
| *Trebouxia* BMP-4 | A09 | Leavitt et al. 2015 | KR913166 |
| *Trebouxia brindabellae* | S13 | del Campo et al. 2010 | FJ626727 |
| *Trebouxia corticola/Trebouxia usneae* | C03 | Friedl et al. 2000 | AJ249573 |
| *Trebouxia crenulata* | A13 | Leavitt et al. 2015 | KR913212 |
| *Trebouxia crespoana* | C01 | Leavitt et al. 2015 | KR913271 |
| *Trebouxia cretacea* | A01 | Leavitt et al. 2015 | KR912355 |
| *Trebouxia cretacea* | A31 | Leavitt et al. 2015 | KR913268 |
| *Trebouxia decolorans* | A33 | Muggia et al. 2014 | KJ754238 |
| *Trebouxia flava* | I03 | Kroken & Taylor 2000 | AF242467 |
| *Trebouxia galapagensis* | C05 | Friedl et al. 2000 | AJ249567 |
| *Trebouxia gelatinosa* | I05 | Bhattacharya et al. 1996 | Z68697 |
| *Trebouxia gigantea* | A38 | Helms et a. 2001 | AJ293789 |
| *Trebouxia higginsiae* | C07 | Friedl et al. 2000 | AJ249574 |
| *Trebouxia impressa/Trebouxia potteri* | I01 | Ruprecht et al. 2012 | JN204778 |
| *Trebouxia impressa/Trebouxia potteri* | I04 | Kroken & Taylor 2000 | AF242469 |
| *Trebouxia incrustata* | A06 | Leavitt et al. 2015 | KR912995 |
| *Trebouxia jamesii* | A03 | Leavitt et al. 2015 | KR912778 |
| *Trebouxia showmanii* | A23 | del Campo et al. 2010 | FJ626734 |
| *Trebouxia simplex* | - | Muggia et al. 2014 | KJ754211 |
| *Trebouxia simplex* | - | Muggia et al. 2014 | KJ754212 |
| *Trebouxia simplex* | - | Muggia et al. 2014 | KJ754213 |
| *Trebouxia simplex* | - | Muggia et al. 2014 | KJ754214 |
| *Trebouxia simplex* | - | Muggia et al. 2014 | KJ754215 |
| *Trebouxia simplex* | - | Muggia et al. 2014 | KJ754216 |
| *Trebouxia simplex* | - | Muggia et al. 2014 | KJ754217 |
| *Trebouxia simplex* | S10 | del Campo et al. 2010 | FJ626735 |
| *Trebouxia solaris* | A35 | Nyati et al. 2014 | AJ969545 |
| *Trebouxia sp.* A02 | A02 | Leavitt et al. 2015 | KR912575 |
| *Trebouxia sp.* A05 | A05 | Leavitt et al. 2015 | KR912914 |
| *Trebouxia sp.* A07 | A07 | Leavitt et al. 2015 | KR913063 |
| *Trebouxia sp.* A08 | A08 | Leavitt et al. 2015 | KR913104 |
| *Trebouxia sp.* A15 (gigantea?) | A15 | Helms et a. 2001 | AJ293790 |
| *Trebouxia sp.* A16 | A16 | Leavitt et al. 2015 | KR913225 |
| *Trebouxia sp.* A17 | A17 | Leavitt et al. 2015 | KR913232 |
| *Trebouxia sp.* A18 | A18 | Leavitt et al. 2015 | KR913237 |
| *Trebouxia sp.* A19 | A19 | Leavitt et al. 2015 | KR913243 |
| *Trebouxia sp.* A20 | A20 | Leavitt et al. 2015 | KR913248 |
| *Trebouxia sp.* A21 | A21 | Leavitt et al. 2015 | KR913253 |
| *Trebouxia sp.* A22 | A22 | Leavitt et al. 2015 | KR913255 |
| *Trebouxia sp.* A24 | A24 | Leavitt et al. 2015 | KR913257 |
| *Trebouxia sp.* A25 | A25 | Leavitt et al. 2015 | KR913259 |
| *Trebouxia sp.* A27 | A27 | Leavitt et al. 2015 | KR913263 |
| *Trebouxia sp.* A28 | A28 | Leavitt et al. 2015 | KR913265 |
| *Trebouxia sp.* A29 | A29 | Leavitt et al. 2015 | KR913266 |
| *Trebouxia sp.* A30 | A30 | Leavitt et al. 2015 | KR913267 |
| *Trebouxia sp.* A32 | A32 | Leavitt et al. 2015 | KR913269 |
| *Trebouxia sp.* A36 | A36 | Nyati et al. 2014 | AJ969540 |
| *Trebouxia sp.* A37 | A37 | Muggia et al. 2014 | KJ754236 |
| *Trebouxia sp.* A43 | A43 | Muggia et al. 2020 | MT127655 |
| *Trebouxia sp.* A44 | A44 | Muggia et al. 2020 | MT127659 |
| *Trebouxia sp.* A45 | A45 | Lindgren et al. 2014 | KJ576675 |
| *Trebouxia sp.* A46 | A46 | Nyati et al. 2014 | AJ969583 |
| *Trebouxia sp.* A47 | A47 | Nyati et al. 2014 | AJ969505 |
| *Trebouxia sp.* A48 | A48 | Nyati et al. 2014 | AJ969587 |
| *Trebouxia sp*. A49 | A49 | Muggia et al. 2020 | MT127660 |
| *Trebouxia sp.* A50 | A50 | Muggia et al. 2014 | KJ754239 |
| *Trebouxia sp.* A51 | A51 | Nyati et al. 2014 | AJ969534 |
| *Trebouxia sp.* C02 | C02 | Leavitt et al. 2015 | KR913285 |
| *Trebouxia sp.* C04 | C04 | Leavitt et al. 2015 | KR913286 |
| *Trebouxia sp.* C06 | C06 | Leavitt et al. 2015 | KR913288 |
| *Trebouxia sp.* C08 | C08 | Muggia et al. 2020 | MT127663 |
| *Trebouxia sp.* C09 | C09 | Muggia et al. 2014 | KJ754198 |
| *Trebouxia sp.* C10 | C10 | Ohmura et al. 2006 | AB177819 |
| *Trebouxia sp.* C11 | C11 | Muggia et al. 2020 | MT127699 |
| *Trebouxia sp.* C12 | C12 | Muggia et al. 2020 | MT127700 |
| *Trebouxia sp.* C13 | C13 | Muggia et al. 2020 | MT127701 |
| *Trebouxia sp.* C14 | C14 | Muggia et al. 2020 | MT127705 |
| *Trebouxia sp.* C15 | C15 | Muggia et al. 2020 | MT127707 |
| *Trebouxia sp.* C16 | C16 | Muggia et al. 2020 | MT127709 |
| *Trebouxia sp.* C17 | C17 | Muggia et al. 2020 | MT127710 |
| *Trebouxia sp.* C18 | C18 | Muggia et al. 2020 | MT127718 |
| *Trebouxia sp.* C19 | C19 | Muggia et al. 2020 | MT127719 |
| *Trebouxia sp.* C20 | C20 | Muggia et al. 2020 | MT127722 |
| *Trebouxia sp.* C21 | C21 | Muggia et al. 2020 | MT127726 |
| *Trebouxia sp.* C22 | C22 | Muggia et al. 2020 | MT127727 |
| *Trebouxia sp.* C23 | C23 | Muggia et al. 2020 | MT127729 |
| *Trebouxia sp*. C24 | C24 | Muggia et al. 2020 | MT127733 |
| *Trebouxia sp.* C25 | C25 | Muggia et al. 2020 | MT127734 |
| *Trebouxia sp.* C26 | C26 | Muggia et al. 2020 | MT127736 |
| *Trebouxia sp.* C27 | C27 | Muggia et al. 2020 | MT127742 |
| *Trebouxia sp.* C28 | C28 | Muggia et al. 2020 | MT127744 |
| *Trebouxia sp.* C29 | C29 | Muggia et al. 2020 | MT127745 |
| *Trebouxia sp.* C30 | C30 | Muggia et al. 2020 | MT127746 |
| *Trebouxia sp.* C31 | C31 | Muggia et al. 2020 | MT127749 |
| *Trebouxia sp*. I02 | I02 | Muggia et al. 2020 | MT127752 |
| *Trebouxia sp.* I06 | I06 | Leavitt et al. 2015 | KR914029 |
| *Trebouxia sp.* I07 | I07 | Leavitt et al. 2015 | KR914035 |
| *Trebouxia sp.* I08 | I08 | Leavitt et al. 2015 | KR914041 |
| *Trebouxia sp.* I09 | I09 | Leavitt et al. 2015 | KR914044 |
| *Trebouxia sp.* I10 | I10 | Leavitt et al. 2015 | KR914047 |
| *Trebouxia sp.* I11 | I11 | Leavitt et al. 2015 | KR914050 |
| *Trebouxia sp.* I12 | I12 | Leavitt et al. 2015 | KR914052 |
| *Trebouxia sp.* I13 | I13 | Leavitt et al. 2015 | KR914054 |
| *Trebouxia sp*. I14 | I14 | Leavitt et al. 2015 | KR914055 |
| *Trebouxia sp.* I15 | I15 | Leavitt et al. 2015 | KR914056 |
| *Trebouxia sp.* I16 | I16 | Muggia et al. 2020 | MT127763 |
| *Trebouxia sp.* I17 | I17 | Muggia et al. 2020 | MT127768 |
| *Trebouxia sp.* I18 | I18 | Muggia et al. 2014 | KJ754203 |
| *Trebouxia sp.* S01 | S01 | Kroken & Taylor 2000 | AF242460 |
| *Trebouxia sp.* S04 | S04 | Leavitt et al. 2015 | KR914113 |
| *Trebouxia sp.* S06 | S06 | Leavitt et al. 2015 | KR914169 |
| *Trebouxia sp.* S09 | S09 | Ruprecht et al. 2012 | GQ375351 |
| *Trebouxia sp.* S12 | S12 | Seifried & Printzen (2009 unpubl.) | GU124701 |
| *Trebouxia sp.* S14 | S14 | Muggia et al. 2014 | KJ754220 |
| *Trebouxia sp.* S15 | S15 | Muggia et al. 2014 | KJ754221 |
| *Trebouxia sp.* S16 | S18 | Sadowska-des et al. 2014 | KJ623947 |
| *Trebouxia sp.* S17 | S19 | Muggia et al. 2014 | KJ754227 |
| *Trebouxia sp.* S18 | S20 | Sadowska-des et al. 2014 | KJ623937 |
| *Trebouxia sp.* S19 | S21 | Perez-Ortega et al. 2012 | JX036273 |
| *Trebouxia sp.* S20 | S22 | Lindgren et al. 2014 | KJ576641 |
| *Trebouxia sp.* S21 | S23 | Lindgren et al. 2014 | KJ576642 |
| *Trebouxia suecica* | S05 | del Campo et al. 2010 | FJ626736 |
| *Trebouxia* TR9 | A39 | del Campo et al. 2013 | GU252197 |
| *Trebouxia vagua* | A04 | Leavitt et al. 2015 | KR913187 |
| *Trebouxia vagua* | A10 | Leavitt et al. 2015 | KR912891 |
| *Trebuxia* URa4 | A12 | Leavitt et al. 2015 | KR913205 |
|  |  |  |  |
