# Supplementary material for: Photobiont Diversity in Lichen Symbioses From Extreme Environments
Source: Front Microbiol. 2022 Mar 29;13:809804. doi: 10.3389/fmicb.2022.809804 (PMC9002315; doi:10.3389/fmicb.2022.809804)
Supplement: Supplementary Table 2 — NCBI accession numbers are reported for the ITS sequences of Trebouxia species-level lineage identified directly from thallus sequencing (thallus ID) and from axenically isolated cultured strains (culture isolates ID). TSB herbarium number is reported for the original lichen samples. [file Data_Sheet_2.DOCX]

| **TSB herbarium number** | **Lichen species** | **Thallus ID** | **Photobiont species from thallus** | **ITS accession (from thallus)** | **Culture isolates ID** | **Isolated photobiont species** | **ITS accession (from isolate)** |
| --- | --- | --- | --- | --- | --- | --- | --- |
| 43176 | *Rhizoplaca melanophthalma* | L2384 | *Trebouxia*A52 | OM275483 | **-** | **-** | **-** |
| 43177 | *Rhizoplaca melanophthalma* | L2385 (*1) | *Trebouxia*A52 | OM275484 | L2907 | *Trebouxia*A52 | OM275533 |
|  |  |  |  |  | L2908 | *Trebouxia*A52 | OM275534 |
|  |  |  |  |  | L2909 | *Trebouxia*A52 | OM275535 |
|  |  |  |  |  | L2910 | *Trebouxia*A52 | OM275536 |
|  |  |  |  |  | L2911 | *Trebouxia*A52 | OM275537 |
|  |  |  |  |  | L2912 | *Trebouxia*A52 | OM275538 |
| 43180 | *Rhizoplaca melanophthalma* | L2388 (*2) | *Trebouxia*A52 | OM275485 | L2901 | *Trebouxia*A52 | OM275527 |
|  |  |  |  |  | L2902 | *Trebouxia*A52 | OM275528 |
|  |  |  |  |  | L2903 | *Trebouxia*A52 | OM275529 |
|  |  |  |  |  | L2904 | *Trebouxia*A02 | OM275530 |
|  |  |  |  |  | L2905 | *Trebouxia*A52 | OM275531 |
|  |  |  |  |  | L2906 | *Trebouxia*A52 | OM275532 |
| 43181 | *Rhizoplaca melanophthalma* | L2389 (*3) | *Trebouxia*A52 | OM275486 | L2913 | *Trebouxia*A52 | OM275539 |
|  |  |  |  |  | L2914 | *Trebouxia*A52 | OM275540 |
|  |  |  |  |  | L2915 | *Trebouxia*A16 | OM275541 |
|  |  |  |  |  | L2916 | *Trebouxia*A52 | OM275542 |
|  |  |  |  |  | L2917 | *Trebouxia vagua*A04 | OM275543 |
|  |  |  |  |  | L2918 | *Trebouxia*A52 | OM275544 |
|  |  |  |  |  | L2919 | *Trebouxia vagua*A04 | OM275545 |

| 43190 | *Rhizoplaca melanophthalma* | L2398 (*4) | *Trebouxia cretacea*A01/A31 | OM275487 | L2920 | *Trebouxia impressa*I04 | OM275546 |
| --- | --- | --- | --- | --- | --- | --- | --- |
|  |  |  |  |  | L2921 | *Trebouxia cretacea*A01/A31 | OM275547 |
|  |  |  |  |  | L2922 | *Trebouxia cretacea*A01/A31 | OM275548 |
|  |  |  |  |  | L2923 | *Trebouxia cretacea*A01/A31 | OM275549 |
|  |  |  |  |  | L2924 | *Trebouxia cretacea*A01/A31 | OM275550 |
| 43192 | *Rhizoplaca melanophthalma* | L2400 (*5) | *Trebouxia*A52 | OM275488 | L2925 | *Trebouxia vagua*A04 | OM275551 |
|  |  |  |  |  | L2926 | *Trebouxia vagua*A04 | OM275552 |
|  |  |  |  |  | L2927 | *Trebouxia cretacea*A01/A31 | OM275553 |
|  |  |  |  |  | L2928 | *Trebouxia cretacea*A01/A31 | OM275554 |
|  |  |  |  |  | L2930 | *Trebouxia*A52 | OM275556 |
| 43214 | *Rhizoplaca melanophthalma* | L2421 (*6) | *Trebouxia*A52 | OM275489 | L2946 | *Trebouxia*A02 | OM275569 |
|  |  |  |  |  | L2947 | *Trebouxia*A02 | OM275570 |
|  |  |  |  |  | L2948 | *Trebouxia*A02 | OM275571 |
|  |  |  |  |  | L3169 | *Trebouxia*A02 | OM275642 |
| 43221 | *Rhizoplaca melanophthalma* | L2428 | *Trebuoxia*A02 | OM275490 | **-** | **-** | **-** |
| 43244 | *Rhizoplaca melanophthalma* | L2452 (*7) | *Trebuoxia*A02 | OM275491 | L2932 | *Trebouxia*A02 | OM275557 |
|  |  |  |  |  | L2933 | *Trebouxia*A02 | OM275558 |
|  |  |  |  |  | L2934 | *Trebouxia*A02 | OM275559 |
|  |  |  |  |  | L2935 | *Trebouxia*A02 | OM275560 |
|  |  |  |  |  | L2936 | *Trebouxia*A02 | OM275561 |
| 43247 | *Rhizoplaca melanophthalma* | L2455 (*8) | **-** | **-** | L2937 | *Trebouxia*A02 | OM275562 |
|  |  |  |  |  | L2938 | *Trebouxia*A02 | OM275563 |
|  |  |  |  |  | L2939 | *Trebouxia*A02 | OM275564 |
|  |  |  |  |  | L2940 | *Trebouxia*A02 | OM275565 |
|  |  |  |  |  | L2941 | *Trebouxia*A02 | OM275566 |
| 43252 | *Rhizoplaca melanophthalma* | L2460 (*42) | *Trebouxia*A02 | OM275492 | L2942 | *Trebouxia*A02 | OM275567 |
| 43297 | *Rhizoplaca melanophthalma* | L2505 | *Trebouxia*A02 | OM275493 | **-** | **-** | **-** |
| 43305 | *Rhizoplaca melanophthalma* | L2513 | *Trebouxia flava*I03 | OM275494 | **-** | **-** | **-** |
| 43134 | *Tephromela atra* | L2545 (*9) | *Trebouxia vagua*A04 | OM275495 | L2963 | *Trebouxia vagua*A04 | OM275585 |
|  |  |  |  |  | L2964 | *Trebouxia vagua*A04 | OM275586 |
| 43140 | *Tephromela atra* | L2551 (*10) | *Trebouxia*A02 | OM275496 | L2977 | *Trebouxia impressa*I04 | OM275597 |
|  |  |  |  |  | L2978 | *Trebouxia vagua*A04 | OM275598 |
|  |  |  |  |  | L3175 | *Trebouxia vagua*A04 | OM275646 |
|  |  |  |  |  | L3176 | *Trebouxia vagua*A04 | OM275647 |
|  |  |  |  |  | L3177 | *Trebouxia vagua*A04 | OM275649 |
| 43149 | *Tephromela atra* | L2560 (*11) | *Trebouxia*A02 | OM275497 | L2956 | *Trebouxia vagua*A10 | OM275579 |
|  |  |  |  |  | L2957 | *Trebouxia vagua*A10 | OM275580 |
|  |  |  |  |  | L2958 | *Trebouxia vagua*A10 | OM275581 |
|  |  |  |  |  | L2979 | *Trebouxia vagua*A10 | OM275599 |
|  |  |  |  |  | L2980 | *Trebouxia vagua*A10 | OM275600 |
| 43150 | *Tephromela atra* | L2561 (*12) | **-** | **-** | L2949 | *Trebouxia vagua*A10 | OM275572 |
|  |  |  |  |  | L2950 | *Trebouxia vagua*A10 | OM275573 |
|  |  |  |  |  | L2951 | *Trebouxia vagua*A10 | OM275574 |
|  |  |  |  |  | L3170 | *Trebouxia vagua*A10 | OM275643 |
| 44448 | *Tephromela atra* | L2567 (*13) | *Trebouxia flava*I03 | OM275498 | L2971 | *Trebouxia impressa*I04 | OM275591 |
|  |  |  |  |  | L2972 | *Trebouxia impressa*I04 | OM275592 |
|  |  |  |  |  | L2973 | *Trebouxia impressa*I04 | OM275593 |
|  |  |  |  |  | L2974 | *Trebouxia impressa*I04 | OM275594 |
|  |  |  |  |  | L2975 | *Trebouxia vagua*A10 | OM275595 |
| 44420 | *Tephromela atra* | L2570 (*14) | *Trebouxia*S02 | OM275499 | L2929 | *Trebouxia flava*I03 | OM275555 |
| 44421 | *Tephromela atra* | L2571 | *Trebouxia*S02 | OM275500 | **-** | **-** |  |

| 44422 | *Tephromela atra* | L2583 (*15) | *Trebouxia impressa*I04 | OM275501 | L3178 | *Trebouxia cretacea*A01/A31 | OM275648 |
| --- | --- | --- | --- | --- | --- | --- | --- |
|  |  |  |  |  | L3179 | *Trebouxia vagua*A04 | OM275650 |
|  |  |  |  |  | L3259 | *Trebouxia impressa*I04 | OM275698 |
| 44423 | *Rhizoplaca melanophthalma* | L2585 (*16) | *Trebouxia*A02 | OM275502 | L2952 | *Trebouxia flava*I03 | OM275575 |
|  |  |  |  |  | L2953 | *Trebouxia*A02 | OM275576 |
|  |  |  |  |  | L2954 | *Trebouxia flava*I03 | OM275577 |
|  |  |  |  |  | L2955 | *Trebouxia flava*I03 | OM275578 |
|  |  |  |  |  | L3167 | *Trebouxia flava*I03 | OM275641 |
| 44424 | *Rhizoplaca melanophthalma* | L2589 (*17) | *Trebouxia*A02 | OM275503 | L2960 | *Trebouxia flava*I03 | OM275582 |
| 44425 | *Rhizoplaca melanophthalma* | L2593 (*18) | *Trebouxia flava*I03 | OM275504 | L2961 | *Trebouxia impressa*I04 | OM275583 |
|  |  |  |  |  | L2962 | *Trebouxia impressa*I04 | OM275584 |
|  |  |  |  |  | L3182 | *Trebouxia impressa*I04 | OM275653 |
|  |  |  |  |  | L3183 | *Trebouxia impressa*I04 | OM275654 |
| 43157 | *Tephromela atra* | L2597 (*19) | ***-*** | **-** | L2943 | *Trebouxia vagua*A04 | OM275568 |
|  |  |  |  |  | L2965 | *Trebouxia vagua*A04 | OM275587 |
|  |  |  |  |  | L2966 | *Trebouxia vagua*A04 | OM275588 |
|  |  |  |  |  | L2967 | *Trebouxia vagua*A04 | OM275589 |
|  |  |  |  |  | L3180 | *Trebouxia vagua*A04 | OM275651 |
|  |  |  |  |  | L3181 | *Trebouxia vagua*A04 | OM275652 |
| 43158 | *Tephromela atra* | L2598 (*20) | *Trebouxia vagua*A10 | OM275505 | L2976 | *Trebouxia vagua*A10 | OM275596 |
| 43159 | *Tephromela atra* | L2599 (*21) | *Trebouxia vagua*A10 | OM275506 | L2970 | *Trebouxia vagua*A10 | OM275590 |
|  |  |  |  |  | L3246 | *Trebouxia vagua*A04 | OM275697 |
| 44426 | *Rhizoplaca melanophthalma* | L2635 (*22) | *Trebouxia impressa*I04 | OM275507 | L3171 | *Trebouxia flava*I03 | OM275644 |
| 44427 | *Rhizoplaca melanophthalma* | L2669 (*23) | *Trebouxia*A08 | OM275508 | L2984 | *Trebouxia*A08 | OM275604 |

| 44428 | *Rhizoplaca melanophthalma* | L2671 (*24) | *Trebouxia*A08 | OM275509 | L3013 | *Trebouxia impressa*I04 | OM275633 |
| --- | --- | --- | --- | --- | --- | --- | --- |
|  |  |  |  |  | L3014 | *Trebouxia impressa*I04 | OM275634 |
|  |  |  |  |  | L3188 | *Trebouxia impressa*I04 | OM275657 |
|  |  |  |  |  | L3189 | *Trebouxia impressa*I04 | OM275658 |
| 44429 | *Rhizoplaca melanophthalma* | L2688 (*43) | *Trebouxia*A02 | OM275510 | L3172 | *Trebouxia*A02 | OM275645 |
| 44430 | *Rhizoplaca melanophthalma* | L2689 (*25) | *Trebouxia cretacea*A01/A31 | OM275511 | L2981 | *Trebouxia cretacea*A01/A31 | OM275601 |
|  |  |  |  |  | L2982 | *Trebouxia cretacea*A01/A31 | OM275602 |
|  |  |  |  |  | L2983 | *Trebouxia cretacea*A01/A31 | OM275603 |
|  |  |  |  |  | L3194 | *Trebouxia cretacea*A01/A31 | OM275663 |
|  |  |  |  |  | L3195 | *Trebouxia cretacea*A01/A31 | OM275664 |
| 44431 | *Rhizoplaca melanophthalma* | L2705 (*26) | *Trebouxia*A02 | OM275512 | L3223 | *Trebouxia*A02 | OM275690 |
|  |  |  |  |  | L3224 | *Trebouxia*A02 | OM275691 |
|  |  |  |  |  | L3225 | *Trebouxia*A02 | OM275692 |
|  |  |  |  |  | L3011 | *Trebouxia*A02 | OM275631 |
| 44432 | *Rhizoplaca melanophthalma* | L2722 (*27) | *Trebouxia flava*I03 | OM275513 | L2985 | *Trebouxia flava* I03 | OM275605 |
|  |  |  |  |  | L2991 | *Trebouxia*A12 | OM275611 |
|  |  |  |  |  | L2992 | *Trebouxia*A12 | OM275612 |
|  |  |  |  |  | L2993 | *Trebouxia cretacea*A01/A31 | OM275613 |
|  |  |  |  |  | L2994 | *Trebouxia cretacea*A01/A31 | OM275614 |
|  |  |  |  |  | L3019 | *Trebouxia cretacea*A01/A31 | OM275639 |
| 44433 | *Rhizoplaca melanophthalma* | L2723 (*28) | *Trebouxia flava*I03 | OM275514 | L3218 | *Trebouxia impressa*I04 | OM275685 |
|  |  |  |  |  | L3219 | *Trebouxia*A02 | OM275686 |
|  |  |  |  |  | L3220 | *Trebouxia*A02 | OM275687 |
|  |  |  |  |  | L3221 | *Trebouxia*A02 | OM275688 |
| 44434 | *Rhizoplaca melanophthalma* | L2724 (*29) | *Trebouxia*A12 | OM275515 | L2989 | *Trebouxia*A12 | OM275609 |
|  |  |  |  |  | L2990 | *Trebouxia*A02 | OM275610 |
| 44435 | *Rhizoplaca melanophthalma* | L2725 (*30) | *Trebouxia flava*I03 | OM275516 | L3012 | *Trebouxia flava*I03 | OM275632 |
|  |  |  |  |  | L3226 | *Trebouxia*A02 | OM275693 |
|  |  |  |  |  | L3227 | *Trebouxia flava*I03 | OM275694 |
|  |  |  |  |  | L3228 | *Trebouxia flava*I03 | OM275695 |
|  |  |  |  |  | L3229 | *Trebouxia flava*I03 | OM275696 |
| 44436 | *Rhizoplaca melanophthalma* | L2732 (*31) | *Trebouxia cretacea*A01/A31 | OM275517 | L3202 | *Trebouxia*A02 | OM275670 |
|  |  |  |  |  | L3203 | *Trebouxia*A02 | OM275671 |
|  |  |  |  |  | L3204 | *Trebouxia*A02 | OM275672 |
|  |  |  |  |  | L3205 | *Trebouxia*A02 | OM275673 |
| 44437 | *Rhizoplaca melanophthalma* | L2733 (*32) | *Trebouxia*A02 | OM275518 | L3206 | *Trebouxia*A02 | OM275674 |
|  |  |  |  |  | L3207 | *Trebouxia*A02 | OM275675 |
|  |  |  |  |  | L3208 | *Trebouxia*A02 | OM275676 |
|  |  |  |  |  | L3209 | *Trebouxia*A02 | OM275677 |
|  |  |  |  |  | L3210 | *Trebouxia*A02 | OM275678 |
|  |  |  |  |  | L3211 | *Trebouxia*A02 | OM275679 |
| 44438 | *Rhizoplaca melanophthalma* | L2734 (*33) | **-** | **-** | L3197 | *Trebouxia*A02 | OM275665 |
|  |  |  |  |  | L3216 | *Trebouxia*A02 | OM275683 |
|  |  |  |  |  | L3217 | *Trebouxia*A02 | OM275684 |
| 44439 | *Rhizoplaca melanophthalma* | L2735 (*34) | *Trebouxia*A02 | OM275519 | L3009 | *Trebouxia*A02 | OM275629 |
|  |  |  |  |  | L3010 | *Trebouxia*A02 | OM275630 |
|  |  |  |  |  | L3222 | *Trebouxia*A02 | OM275689 |

| 44440 | *Rhizoplaca melanophthalma* | L2787 (*35) | *Trebouxia impressa*I04 | OM275520 | L3003 | *Trebouxia impressa*I04 | OM275623 |
| --- | --- | --- | --- | --- | --- | --- | --- |
|  |  |  |  |  | L3004 | *Trebouxia impressa*I04 | OM275624 |
|  |  |  |  |  | L3005 | *Trebouxia*A02 | OM275625 |
|  |  |  |  |  | L3006 | *Trebouxia impressa*I04 | OM275626 |
|  |  |  |  |  | L3007 | *Trebouxia impressa*I04 | OM275627 |
|  |  |  |  |  | L3186 | *Trebouxia impressa*I04 | OM275655 |
|  |  |  |  |  | L3187 | *Trebouxia impressa*I04 | OM275656 |
| 44441 | *Rhizoplaca melanophthalma* | L2796 (*36) | *Trebouxia*A12 | OM275521 | L3015 | *Trebouxia*A02 | OM275635 |
|  |  |  |  |  | L3016 | *Trebouxia impressa*I04 | OM275636 |
|  |  |  |  |  | L3017 | *Trebouxia impressa*I04 | OM275637 |
|  |  |  |  |  | L3212 | *Trebouxia impressa*I04 | OM275680 |
|  |  |  |  |  | L3213 | *Trebouxia*A02 | OM275681 |
| 44443 | *Rhizoplaca melanophthalma* | L2803 (*37) | *Trebouxia vagua* A04 | OM275523 | L2997 | *Trebuxia*A02 | OM275617 |
|  |  |  |  |  | L2998 | *Trebouxia impressa*I04 | OM275618 |
|  |  |  |  |  | L2999 | *Trebuxia*A02 | OM275619 |
|  |  |  |  |  | L3000 | *Trebuxia*A02 | OM275620 |
|  |  |  |  |  | L3008 | *Trebuxia*A02 | OM275628 |
| 44442 | *Rhizoplaca melanophthalma* | L2802 (*38) | *Trebouxia*A02 | OM275522 | L3199 | *Trebouxia*A02 | OM275667 |
|  |  |  |  |  | L3200 | *Trebouxia*A02 | OM275668 |
|  |  |  |  |  | L3201 | *Trebouxia*A02 | OM275669 |
| 44444 | *Rhizoplaca melanophthalma* | L2824 (*39) | *Trebouxia*A02 | OM275524 | L2986 | *Trebouxia cretacea*A01/A31 | OM275606 |
|  |  |  |  |  | L2987 | *Trebouxia*A02 | OM275607 |
|  |  |  |  |  | L2988 | *Trebouxia*A02 | OM275608 |
|  |  |  |  |  | L3020 | *Trebouxia*A02 | OM275640 |
|  |  |  |  |  | L3214 | *Trebouxia*A02 | OM275682 |
| 44445 | *Rhizoplaca melanophthalma* | L2825 | **-** | **-** | L3198 | *Trebouxia*A02 | OM275666 |
| 44446 | *Rhizoplaca melanophthalma* | L2826 (*40) | *Trebouxia impressa*I04 | OM275525 | L3001 | *Trebouxia*A02 | OM275621 |
|  |  |  |  |  | L3002 | *Trebouxia*A02 | OM275622 |
| 44447 | *Rhizoplaca melanophthalma* | L2827 (*41) | *Trebouxia*A02 | OM275526 | L2995 | *Trebouxia cretacea*A01/A31 | OM275615 |
|  |  |  |  |  | L2996 | *Trebouxia cretacea*A01/A31 | OM275616 |
|  |  |  |  |  | L3018 | *Trebouxia*A02 | OM275638 |
|  |  |  |  |  | L3190 | *Trebouxia*A02 | OM275659 |
|  |  |  |  |  | L3191 | *Trebouxia*A02 | OM275660 |
|  |  |  |  |  | L3192 | *Trebouxia*A02 | OM275661 |
|  |  |  |  |  | L3193 | *Trebouxia*A02 | OM275662 |
| 42617 | *Tephromela atra* | L3272 | *Trebouxia*S02 | OM275699 | **-** | **-** | **-** |
| 42618 | *Tephromela atra* | L3273 | *Trebouxia*S02 | OM275700 | **-** | **-** | **-** |
| 42625 | *Tephromela atra* | L3280 | *Trebouxia*S02 | OM275701 | **-** | **-** | **-** |
| 42807 | *Rhizoplaca melanophthalma* | L3287 (*44) | *Trebouxia*S02 | OM275702 | L4118 | *Trebouxia flava*I03 | OM275762 |
|  |  |  |  |  | L4119 | *Trebouxia flava*I03 | OM275763 |
| 42813 | *Rhizoplaca melanophthalma* | L3293 | *Trebouxia flava*I03 | OM275703 | **-** | **-** | **-** |
| 42632 | *Tephromela atra* | L3314 (*45) | *Trebouxia flava*I03 | OM275704 | L4120 | *Trebouxia impressa*I04 | OM275764 |
|  |  |  |  |  | L4121 | *Trebouxia impressa*I04 | OM275765 |
| 42635 | *Tephromela atra* | L3317 | *Trebouxia impressa*I04 | OM275705 | **-** | **-** | **-** |
| 42837 | *Rhizoplaca cf. melanophthalma* | L3335 (*46) | *Trebouxia*S02 | OM275706 | L4127 | *Trebouxia flava*I03 | OM275766 |
| 42838 | *Rhizoplaca cf. melanophthalma* | L3336 (*47) | *Trebouxia impressa*I04 | OM275707 | L4129 | *Trebouxia impressa*I04 | OM275767 |
|  |  |  |  |  | L4130 | *Trebouxia impressa*I04 | OM275768 |
| 42841 | *Rhizoplaca cf. melanophthalma* | L3340 | *Trebouxia flava*I03 | OM275708 | **-** | **-** | **-** |
| 42651 | *Tephromela atra* | L3352 | *Trebouxia*S02 | OM275709 | **-** | **-** | **-** |
| 42657 | *Tephromela atra* | L3358 | *Trebouxia flava*I03 | OM275710 | **-** | **-** | **-** |
| 42863 | *Rhizoplaca cf. melanophthalma* | L3365 (*48) | *Trebouxia impressa*I04 | OM275711 | L3887 | *Trebouxia impressa*I04 | OM275751 |
|  |  |  |  |  | L3888 | *Trebouxia impressa*I04 | OM275752 |
| 42675 | *Tephromela atra* | L3396 | *Trebouxia*S02 | OM275712 | **-** | **-** | **-** |
| 42677 | *Tephromela atra* | L3398 | *Trebouxia*S02 | OM275713 | **-** | **-** | **-** |
| 42683 | *Tephromela atra* | L3404 (*49) | *Trebouxia*S02 | OM275714 | L4138 | *Trebouxia impressa*I04 | OM275769 |
| 42684 | *Tephromela atra* | L3405 | *Trebouxia*S02 | OM275715 | **-** | **-** | **-** |
| 42880 | *Rhizoplaca cf. melanophthalma* | L3419 (*50) | *Trebouxia flava*I03 | OM275716 | L3902 | *Trebouxia flava*I03 | OM275753 |
|  |  |  |  |  | L3904 | *Trebouxia flava*I03 | OM275754 |
| 42883 | *Rhizoplaca cf. melanophthalma* | L3422 (*51) | *Trebouxia flava*I03 | OM275717 | L3910 | *Trebouxia flava*I03 | OM275755 |
| 42889 | *Rhizoplaca cf. melanophthalma* | L3438 | - | - | L3913 | *Trebouxia flava*I03 | OM275756 |
| 42900 | *Rhizoplaca cf. melanophthalma* | L3439 | *Trebouxia flava*I03 | OM275718 | **-** | **-** | **-** |
| 42901 | *Rhizoplaca cf. melanophthalma* | L3440 (*52) | *Trebouxia impressa*I04 | OM275719 | L3915 | *Trebouxia flava*I03 | OM275757 |
| 42700 | *Tephromela atra* | L3470 | *Trebouxia*S02 | OM275720 | **-** | **-** | **-** |
| 42701 | *Tephromela atra* | L3471 | *Trebouxia*S02 | OM275721 | **-** | **-** | **-** |
| 42704 | *Tephromela atra* | L3474 | *Trebouxia impressa*I04 | OM275723 | **-** | **-** | **-** |
| 42702 | *Tephromela atra* | L3472 (*53) | *Trebouxia*S02 | OM275722 | L4144 | *Trebouxia impressa*I04 | OM275770 |
|  |  |  |  |  | L4145 | *Trebouxia flava*I03 | OM275771 |
| 42929 | *Rhizoplaca cf. melanophthalma* | L3481 | *Trebouxia flava*I03 | OM275724 | **-** | **-** | **-** |
| 42932 | *Rhizoplaca cf. melanophthalma* | L3484 | *Trebouxia flava*I03 | OM275725 | **-** | **-** | **-** |
| 42944 | *Rhizoplaca cf. melanophthalma* | L3496 | *Trebouxia impressa*I04 | OM275726 | **-** | **-** | **-** |
| 42945 | *Rhizoplaca cf. melanophthalma* | L3497 | *Trebouxia impressa*I04 | OM275727 | **-** | **-** | **-** |
| 42714 | *Tephromela atra* | L3523 (*54) | *Trebouxia flava*I03 | OM275728 | L4152 | *Trebouxia impressa*I04 | OM275772 |
| 42727 | *Tephromela atra* | L3536 | *Trebouxia incrustata*A06 | OM275729 | **-** | **-** | **-** |
| 42969 | *Rhizoplaca cf. melanophthalma* | L3538 (*55) | *Trebouxia flava*I03 | OM275730 | L4153 | *Trebouxia flava*I03 | OM275773 |
| 42971 | *Rhizoplaca cf. melanophthalma* | L3540 | **-** | - | L4154 | *Trebouxia flava*I03 | OM275774 |
| 42728 | *Tephromela atra* | L3555 | *Trebouxia impressa*I04 | OM275731 | **-** | **-** | **-** |
| 42731 | *Tephromela atra* | L3559 | *Trebouxia impressa*I04 | OM275732 | **-** | **-** | **-** |
| 42986 | *Rhizoplaca cf. melanophthalma* | L3564 | *Trebouxia flava*I03 | OM275733 | **-** | **-** | **-** |
| 42991 | *Rhizoplaca cf. melanophthalma* | L3569 | *Trebouxia impressa*I04 | OM275734 | **-** | **-** | **-** |
| 42994 | *Rhizoplaca cf. melanophthalma* | L3572 | *Trebouxia flava*I03 | OM275735 | **-** | **-** | **-** |
| 42998 | *Rhizoplaca cf. melanophthalma* | L3576 (*56) | *Trebouxia impressa*I04 | OM275736 | L4155 | *Trebouxia impressa*I04 | OM275775 |
| 42998 | *Rhizoplaca cf. melanophthalma* | L3577 (*57) | *Trebouxia impressa*I04 | OM275737 | L4156 | *Trebouxia impressa*I04 | OM275776 |
| 43015 | *Rhizoplaca cf. melanophthalma* | L3594 (*58) | *Trebouxia impressa*I04 | OM275738 | L4159 | *Trebouxia impressa*I04 | OM275777 |
|  |  |  |  |  | L4160 | *Trebouxia impressa*I04 | OM275778 |
| 43037 | *Rhizoplaca cf. melanophthalma* | L3616 | **-** | **-** | L4162 | *Trebouxia flava*I03 | OM275779 |
| 43037 | *Rhizoplaca cf. melanophthalma* | L3617 | **-** | **-** | L4163 | *Trebouxia flava*I03 | OM275780 |
|  |  |  |  |  | L4164 | *Trebouxia flava*I03 | OM275781 |
| 42743 | *Tephromela atra* | L3648 | *Trebouxia*S02 | OM275739 | **-** | **-** | **-** |
| 43057 | *Rhizoplaca cf. melanophthalma* | L3653 (*59) | *Trebouxia impressa*I04 | OM275740 | L3951 | *Trebouxia impressa*I04 | OM275758 |
| 43059 | *Rhizoplaca cf. melanophthalma* | L3655 (*60) | **-** | **-** | L3953 | *Trebouxia flava*I03 | OM275760 |
|  |  |  |  |  | L3952 | *Trebouxia impressa*I04 | OM275759 |
|  |  |  |  |  | L4172 | *Trebouxia flava*I03 | OM275782 |
| 42749 | *Tephromela atra* | L3681 (*61) | *Trebouxia impressa*I04 | OM275741 | L4176 | *Trebouxia impressa*I04 | OM275783 |
| 42760 | *Tephromela atra* | L3692 | *Trebouxia impressa*I04 | OM275742 | **-** | ***-*** | **-** |
| 42764 | *Tephromela atra* | L3696 (*62) | **-** | **-** | L4178 | *Trebouxia impressa*I04 | OM275784 |
|  |  |  |  |  | L4180 | *Trebouxia impressa*I04 | OM275785 |
| 42765 | *Tephromela atra* | L3697 (*63) | *Trebouxia*S02 | OM275743 | L4181 | *Trebouxia*S02 | OM275786 |
|  |  |  |  |  | L4183 | *Trebouxia impressa*I04 | OM275787 |
| 42778 | *Tephromela atra* | L3710 (*64) | *Trebouxia*S02 | OM275744 | L4190 | *Trebouxia impressa*I04 | OM275788 |
| 42788 | *Tephromela atra* | L3720 | *Trebouxia*S02 | OM275745 | **-** | **-** | **-** |
| 42790 | *Tephromela atra* | L3722 (*65) | *Trebouxia flava*I03 | OM275746 | L4193 | *Trebouxia impressa*I04 | OM275789 |
|  |  |  |  |  | L4194 | *Trebouxia impressa*I04 | OM275790 |
| 43083 | *Rhizoplaca cf. melanophthalma* | L3724 (*66) | *Trebouxia impressa*I04 | OM275747 | L4196 | *Trebouxia flava*I03 | OM275791 |
| 43084 | *Rhizoplaca cf. melanophthalma* | L3725 (*67) | *Trebouxia flava*I03 | OM275748 | L3955 | *Trebouxia flava*I03 | OM275761 |
| 42791 | *Tephromela atra* | L3820 | *Trebouxia*S02 | OM275749 | **-** | **-** | **-** |
| 42792 | *Tephromela atra* | L3821 | *Trebouxia*S02 | OM275750 | **-** | **-** | **-** |
